# Trophic Position of the White Worm (*Enchytraeus albidus*) in the Context of Digestive Enzyme Genes Revealed by Transcriptomics Analysis

**DOI:** 10.3390/ijms25094685

**Published:** 2024-04-25

**Authors:** Łukasz Gajda, Agata Daszkowska-Golec, Piotr Świątek

**Affiliations:** Institute of Biology, Biotechnology and Environmental Protection, Faculty of Natural Sciences, University of Silesia in Katowice, 9 Bankowa St., 40-007 Katowice, Poland; lgajda@us.edu.pl (Ł.G.); agata.daszkowska@us.edu.pl (A.D.-G.)

**Keywords:** potworms, decomposers, transcriptome, cellulase, digestive lysozyme, COI-monohaplotype culture

## Abstract

To assess the impact of Enchytraeidae (potworms) on the functioning of the decomposer system, knowledge of the feeding preferences of enchytraeid species is required. Different food preferences can be explained by variations in enzymatic activities among different enchytraeid species, as there are no significant differences in the morphology or anatomy of their alimentary tracts. However, it is crucial to distinguish between the contribution of microbial enzymes and the animal’s digestive capacity. Here, we computationally analyzed the endogenous digestive enzyme genes in *Enchytraeus albidus*. The analysis was based on RNA-Seq of COI-monohaplotype culture (PL-A strain) specimens, utilizing transcriptome profiling to determine the trophic position of the species. We also corroborated the results obtained using transcriptomics data from genetically heterogeneous freeze-tolerant strains. Our results revealed that *E*. *albidus* expresses a wide range of glycosidases, including GH9 cellulases and a specific digestive SH3b-domain-containing i-type lysozyme, previously described in the earthworm *Eisenia andrei*. Therefore, *E*. *albidus* combines traits of both primary decomposers (primary saprophytophages) and secondary decomposers (sapro-microphytophages/microbivores) and can be defined as an intermediate decomposer. Based on assemblies of publicly available RNA-Seq reads, we found close homologs for these cellulases and i-type lysozymes in various clitellate taxa, including Crassiclitellata and Enchytraeidae.

## 1. Introduction

The 1975 article by J. M. Anderson, “The Enigma of Soil Animal Species Diversity”, highlighted the high species richness found in soils and emphasized the unknown mechanisms contributing to this diversity [1]. Despite several new hypotheses, the mechanisms driving species richness in soils have remained largely elusive [2,3]. The relationship between ecosystem characteristics and the number of trophic levels in food webs has been debated, with some studies suggesting that the number of trophic levels increases with productivity and resource availability [4], while others propose that nutrient-poor systems have more trophic levels due to a large number of interactions between species [5]. Over the past decade, researchers have also hypothesized that the high species richness observed in small quantities of soil is related to the high heterogeneity found at very fine scales within the soil [2]. However, the enigma of how large numbers of soil animal species occupying the same trophic level, such as decomposers, can coexist in one food web remains an open question. Traditional research methods often provide only limited information on feeding, leaving the trophic status of many soil invertebrate groups uncertain or theoretical [6,7,8]. Feeding is a complex process that involves food choice, ingestion, digestion, assimilation, and retention. Traditional research methods, which include direct observation of feeding behavior, gut content analyses, enzymatic analyses of whole-body homogenates, cultivation on different nutrient sources, or choice tests, typically address only one or a few component processes and are unable to provide comprehensive information about the exact source and components digested and assimilated from the ingested food bolus [7,9]. Significant advancements in the understanding of the diets and trophic interactions of soil animals in recent years have been made possible through more sophisticated methods such as stable isotope analysis [9]. This technique provides estimates of the retention of atoms from basal food resources and allows for the indication of the trophic level of the analyzed group of animals in the food web. However, bulk natural stable isotopes provide only rough information about the basal resources used by the analyzed animals, rarely allowing for the reconstruction of species-specific feeding interactions in soil [7]. Distinguishing between bacterial and fungal feeding, as well as feeding on different taxa of microorganisms, is challenging and often impossible using stable isotope analysis alone [8,10]. Moreover, several ontogenetic, physiological, and biochemical factors can affect the isotopic composition of animal tissues [6,11]. Another challenge is the dietary flexibility exhibited by many soil animals, which can vary depending on available food sources and may result in these animals operating on more than one trophic level [8,12]. Furthermore, the contribution of microbial enzymatic apparatus to the invertebrate digestion process cannot be overlooked. The enzyme activity of microbiota or food-associated microorganisms can significantly affect the host’s digestive capabilities [13,14]. Therefore, while stable isotope analysis is currently a leading method in trophic ecology studies, it should be used in conjunction with other complementary approaches, given its limitations. Recently, these combined multi-methodological approaches have successfully revealed the multidimensional trophic niche of springtails (Collembola) [7].

Among the major groups of soil invertebrates, Enchytraeidae, also known as potworms, are no exception when it comes to the uncertainty of their trophic position. Despite being a widely distributed family of small annelids, their trophic status within the soil food web remains unsolved, even after several studies have used stable isotope analysis [11,15,16,17]. It is still unclear whether they should be classified as primary or secondary decomposers. Detailed studies on the food preferences of enchytraeids have only been conducted in a few species [18]. The conclusions drawn from these findings are also limited by the high level of cryptic diversity within the family [19,20,21], as cryptic species may differ in their specific ecological and physiological properties [22,23].

Enchytraeids share the general body plan of oligochaetes and represent a relatively simple and uniform group [24,25]. There are no significant differences in the anatomy of the alimentary tract or highly specialized morphological structures that could clearly indicate the feeding strategy of most potworm species (but cf. *Aspidodrilus kelsalli* or *Pelmatodrilus planariformis* [26]; these two unique species with some flattened body regions are ectocommensals that have adapted to living on earthworms). Different food preferences could be explained by varying enzymatic activities among different enchytraeid species. However, this hypothesis requires support from genetic methods to investigate the endogenous expression of digestive enzyme genes and distinguish the contribution of the microbiota to this process. Traditional biochemical assays are not sufficient for this purpose, as it is challenging to separate enzyme activity originating from the animal itself from activity related to exogenous sources, such as microbiota or food-associated microorganisms [8].

An alternative and more sophisticated approach to traditional biochemical methods in trophic ecology studies, which complements stable isotope analysis, involves the use of RNA sequencing and transcriptome profiling. Transcriptomics provides access to transcriptome-wide gene expression data, enabling the characterization of an organism’s limitations and capacities for various traits [27], including the repertoire of digestive enzyme genes. Although RNA-Seq is commonly used to predict the digestive capacity of economically important species of crustaceans [28,29,30], fish [31], and insects [32,33], this approach has not been widely adopted in trophic ecology studies. Despite using other molecular methods, such as molecular gut content or meta-barcoding microbiome analyses to understand better the trophic links between species and their diets in soil food webs, the potential of RNA-Seq in this field remains largely untapped.

The white worm, *Enchytraeus albidus*, is an economically and scientifically important species of Enchytraeidae. It can be found in both terrestrial and marine littoral habitats [20]. To date, *E*. *albidus* or any other member of the Enchytraeidae has not been the subject of molecular studies regarding its digestive capacity. In the last published review dedicated to the food preferences of enchytraeids [18], a classification of trophic types was proposed for the most commonly studied genera in relation to food preferences and feeding behavior. *Enchytraeus* spp., including *E*. *albidus*, were assigned to the secondary decomposer group. In the present study, we determined the trophic position of *E*. *albidus* based on RNA-Seq data. We obtained raw reads and performed de novo transcriptome assembly for the *E*. *albidus* PL-A strain originating from a COI-monohaplotype culture. We conducted a transcriptome screening, identified the expressed genes involved in digestive enzyme production in *E*. *albidus*, and performed in silico characterization of the sequences. Moreover, we compared and cross-checked the obtained data with transcriptomics data related to the freeze-tolerant German (G) and Greenlandic (N) strains of *E*. *albidus* [34]. Given that primary decomposers are species that primarily feed on litter material that is little colonized by microorganisms, while secondary decomposers mainly feed on microorganisms and/or plant residues that are partially degraded due to microbial activity, we tested the following two hypotheses: (1) *E*. *albidus* does not exhibit endogenous expression of the enzyme genes from the cellulase group, and (2) *E*. *albidus* exhibits endogenous expression of digestive enzyme genes involved in the digestion of bacteria or fungi (e.g., peptidoglycan hydrolases or chitinases). These hypotheses, consistent with the last review’s postulation that *E*. *albidus* belongs to the secondary decomposer group, were confronted with the repertoire of digestive enzyme genes in this species, as revealed by transcriptomics data.

## 2. Results

### 2.1. RNA-Seq, Transcriptome Assembly, and Annotation Results

To decipher the genes expressed and responsible for digestive enzyme production, and given the absence of a reference genome for *E*. *albidus*, we conducted transcriptome sequencing using the RNA-Seq method and performed de novo transcriptome assembly. RNA sequencing was performed on a single sample, comprising four PL-A strain specimens of *E*. *albidus* originating from a single cocoon. A total of 118,210,442 reads were generated, resulting in a cumulative read base of 17.8 gigabases (Gb). The GC content of the raw data was determined to be 44.96%. Furthermore, quality assessment indicated that the percentage of bases with a Phred quality score ≥ 30 (Q30) was 93.42%, while the percentage of bases with a Phred quality score ≥ 20 (Q20) was 97.57%.

As a technical side note pertaining to the quality assessment of RNA samples designated for RNA-Seq, with potential relevance for readers, it should be mentioned that in *E*. *albidus* the 28S ribosomal RNA undergoes fragmentation into two subparts under heat-denaturing conditions due to a so-called hidden break. Consequently, when analyzing the integrity of rRNA, samples exhibited an atypical profile in the Bioanalyzer electropherogram, characterized by a nearly dominant peak at the 18S position and the absence of a typical peak at the 28S position, resulting in a low rRNA ratio (e.g., 0.1) (for more details, see [35,36]). Nonetheless, as described above, the generated reads were of good quality. 

The BUSCO assessment of de novo-assembled transcriptomes for enchytraeid species revealed that *E. albidus* is currently the only species with a transcriptome in the Sequence Read Archive (SRA) that can be considered complete (see Table 1). A comparison of KEGG-annotated transcriptomes for available *E*. *albidus* strains (refer to Table 2) demonstrates that, despite a roughly 30% difference in the number of assembled sequences, our PL-A strain transcriptome, derived from a single run, exhibits a striking similarity in the count of assigned non-redundant KOs (KEGG Orthology identifiers) for metabolic enzymes when compared to the other transcriptomes. However, it should be noted that the lower number of assembled sequences can be attributed to the high genetic homogeneity of our sample, as it was derived from a pure COI-monohaplotype culture, as well as the much lower number of specimens used for RNA-Seq library preparation.

### 2.2. Integrative Annotation of Glycosidase Genes in E. albidus Strains

Using microbial-decontaminated data for our COI-monohaplotype PL-A specimens, and corroborated by information from two intraspecifically heterogeneous freeze-tolerant strains of *E*. *albidus*, we identified over 1900 functional orthologs for metabolic enzymes. From these orthologs, around 40 KO identifiers were assigned to glycosidases by KEGG-GhostKOALA. Additional glycoside hydrolase candidates (i.e., lysozyme and mannan endo-1,4-β-mannosidases) for digestive enzyme genes that were unannotated in the initial GhostKOALA dataset were identified through PANNZER2 and KofamKOALA annotations using adjusted thresholds. Collectively, we pinpointed 30 digestive gene candidates encoding glycosidases and assessed the number of unique sequence variants for them across *E*. *albidus* strains (Table 3). These selected expressed genes could be further roughly grouped into (1) starch- and glycogen-digesting enzymes (α-amylase I/II, maltase-glucoamylase, maltase-glucoamylase intestinal-like isoform/intestinal-like isoform X2), (2) cellulose- and lichenan-digesting enzymes (endo-β-1,4-glucanase I/II, endo-1,3(4)-β-glucanase), (3) chitin-digesting enzymes (chitinase I/II, di-N-acetylchitobiase/di-N-acetylchitobiase isoform X1, and formally lysozyme, which is mainly a peptidoglycan-degrading enzyme), (4) xylan-digesting enzymes (β-glucosidase/xylosidase I–V), and (5) other specific carbohydrate-digesting enzymes (β-galactosidase, β-glucuronidase, α-L-fucosidase I–IV, β-mannosidase, and mannan endo-1,4-β-mannosidase I–IV). It is worth mentioning that no endogenous sequence for α,α-trehalase (α,α-trehalose glucohydrolase, EC 3.2.1.28) was found (only microbial) in the transcriptomes of *E*. *albidus* and other enchytraeid species, which suggests that enchytraeids lack this enzyme, similar to earthworms [37,38].

### 2.3. Integrative Annotation of Protease Genes in E. albidus Strains

Sequence analysis and annotation of *E*. *albidus* transcriptomics data revealed that enchytraeids do not have close homologs of classical trypsin and chymotrypsin enzymes, similar to earthworms [49]. Earthworms possess proteases with trypsin-like and chymotrypsin-like activities, which are involved in the digestion of protein and peptides in food and are mainly localized in the crop, gizzard, and anterior intestine [50,51,52]. These serine proteases, collectively known as lumbrokinases, exhibit fibrinolytic activity and relatively broad substrate specificities [50]. Some of these enzymes can be in glycosylated form [53]. 

Based on the transcriptomics data for *E*. *albidus*, we identified at least four fibrinolytic serine protease genes for which the transcripts were initially KEGG-annotated as trypsin, chymotrypsin, and elastase sequences (Table 4). These fibrinolytic serine proteases share significant identity and similarity with sequences of fibrinolytic enzymes from enchytraeid *Enchytraeus japonensis*, as well as earthworms’ lumbrokinases, as cataloged in GenBank. In *E*. *albidus*, these serine proteases constitute a related protein cluster, presenting sequence and structural parallels that complicate precise BLASTp identification of the potential single closest homolog, at least outside the taxonomic family. Moreover, we identified ten different genes comprising a total of 52 unigenes for carboxypeptidase A/B-like (EC 3.4.17.1; EC 3.4.17.2), and three different genes comprising a total of 14 unigenes for aminopeptidase N (EC 3.4.11.2), shared across the *E*. *albidus* strains (Table 5).

### 2.4. Integrative Annotation of Digestive Lipases in E. albidus Strains

Before nutritional fat can be transported within the body for storage in adipose tissues or direct energy production, it must first undergo hydrolysis by lipolytic enzymes [54]. We identified four candidates for bile salt-stimulated lipase (CEL) genes and one distinct gene candidate for digestive secretory phospholipase A2. The identified lipolytic enzyme genes are presented in Table 6. Among the four expressed CEL genes identified in *E*. *albidus*, bile salt-stimulated lipase IV was not recovered from the assembled transcripts for the PL-A strain. Nonetheless, a BLASTn search on the raw reads (SRX19982531), using the German strain sequence as a query, confirmed the presence of a short fragment (70 amino acids) of identical C-terminal end of the lipase in the data. This indicates that the gene is indeed expressed in the PL-A strain but was not recovered in subsequent steps of transcriptome assembly and protein prediction.

### 2.5. Phylogenetic Analysis of Selected Digestive Enzymes

The collected data (Supplementary Data S1), including transcriptomics data with varying sequencing depths and BUSCO completeness, enabled us to conduct an analysis and construct phylogenetic trees for putative cellulase (endo-β-1,4-glucanase, EC 3.2.1.4) and digestive i-type lysozyme proteins identified in *E*. *albidus* and among other members of Clitellata. This robust dataset underpins our phylogenetic inferences, providing insights into the evolutionary relationships of these enzymes.

#### 2.5.1. Phylogenetic Analysis of Cellulases (Endo-β-1,4-Glucanases)

**Figure 1:**
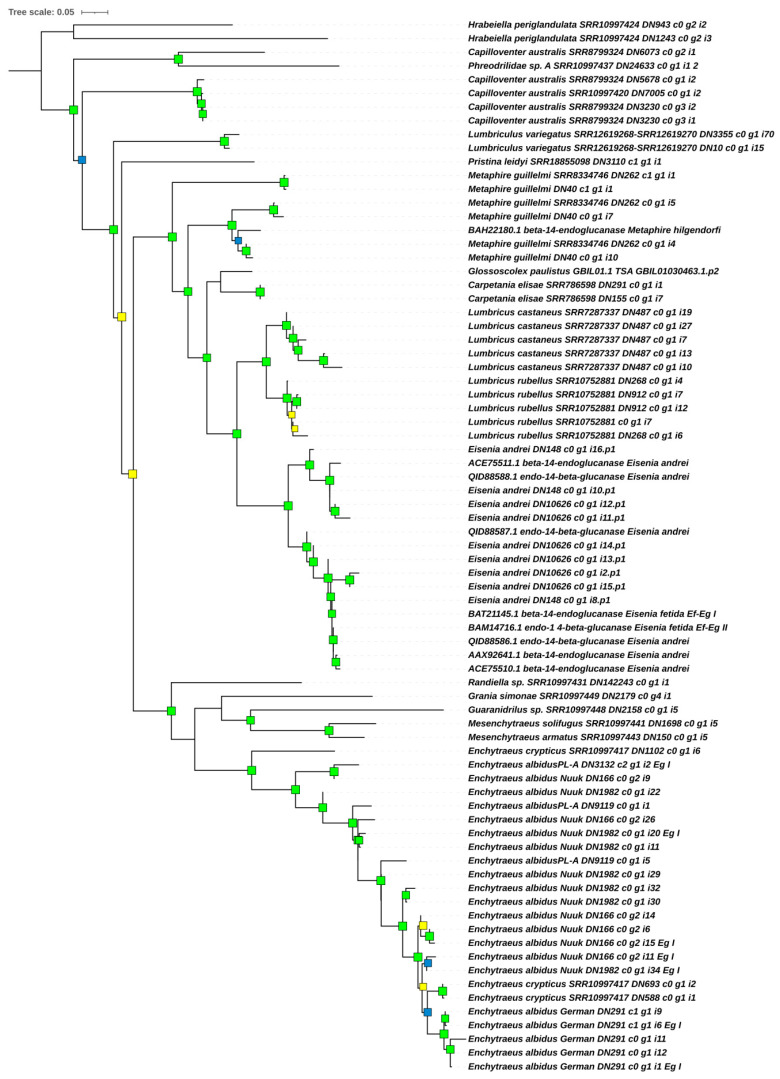
Best-scoring maximum likelihood tree (lnL = −11,983.127) resulting from the analysis of mature amino acid sequences of the putative endo-β-1,4-glucanase I/II proteins in Clitellata. Most sequences used in the analysis were retrieved from SRA transcriptomics data assembled in Trinity. Sequences with identifiers prefixed by an accession number were sourced from GenBank database. Details of the other sources, used for acquiring additional transcriptomics data and included in the phylogenetic analysis, can be found in Supplementary Table S3. Green squares denote branches with both SH-aLRT and UFBoot values (if ≥70) at the respective nodes. Yellow squares indicate support values (if ≥70) only for UFBoot, while blue squares indicate support values (if ≥70) only for SH-aLRT. The tree was rooted at the terrestrial polychaete *Hrabeiella periglandulata*.

Reciprocal BLASTp analyses of endo-β-1,4-glucanase I/II protein sequences primarily obtained from Clitellata revealed significant similarity to previously characterized and closely related cellulases (GH9 family) from earthworms such as *Metaphire hilgendorfi* [40] and *Eisenia* spp. [41,42]. All hits to these sequences had an E-value of 0, indicating a high-quality match (Supplementary Table S1). Additionally, we identified two closely homologous cellulases from the transcriptomics data of the terrestrial polychaete *Hrabeiella periglandulata*. This species is the sole representative of Hrabeiellidae, and along with the polychaete *Aeolosoma* is considered to form a sister group to Clitellata in the species phylogeny of Annelida [55]. After rooting the tree at *H*. *periglandulata*, our phylogenetic analysis of endo-β-1,4-glucanase I/II proteins (Figure 1) indicated that *Capilloventer australis* and Phreodrilidae sp. cellulases diverged from a shared ancestor. Notably, two distinct but homologous cellulase genes have been identified in *C*. *australis*. Furthermore, the *Capilloventer*–Phreodrilidae cluster was found to be a sister to the second paralogous protein variants from *C*. *australis* plus all remaining clitellate sequences. Within the remaining Clitellata, two main clades were recovered with high support. The first one contains a single sequence from the lumbriculid *Lumbriculus variegatus*. In the second, a single representative of Naididae in our analysis—*Pristina leydyi*—was recovered as a sister to Crassiclitellata (represented by earthworm species) plus Enchytraeidae with *Randiella*. The Crassiclitellata cluster was highly resolved, but its evolutionary history appears complex, as indicated by an independent endoglucanase duplication event in the most basally placed *Metaphire guillelmi*. This paralogous copy was recovered as a sister to all other earthworm endoglucanases, including the remaining *Metaphire* sequences.

The second main clade comprised all Enchytraeidae proteins sister to a single cellulase from *Randiella*, with high support. The Enchytraeidae cluster was mostly well resolved. Within Enchytraeidae, two subclusters were recovered. In the first, sequences from *Grania*, *Guaranidrilus*, and *Mesenchytraeus* spp. were grouped. In the second, proteins from *Enchytraeus albidus* and *E*. *crypticus* were grouped together in a manner discordant with species phylogeny. A paralogous sequence from *E*. *crypticus* was recovered as a sister to all other proteins from *E*. *albidus* and *E*. *crypticus*. The remaining *E*. *crypticus* sequences were recovered as nested within *E. albidus*. This may suggest incomplete lineage sorting, as vertical gene flow between those species is unlikely due to physical reproductive barriers, although horizontal gene transfer for *E*. *crypticus* was reported [56].

#### 2.5.2. Phylogenetic Analysis of Digestive i-Type Lysozyme

**Figure 2:**
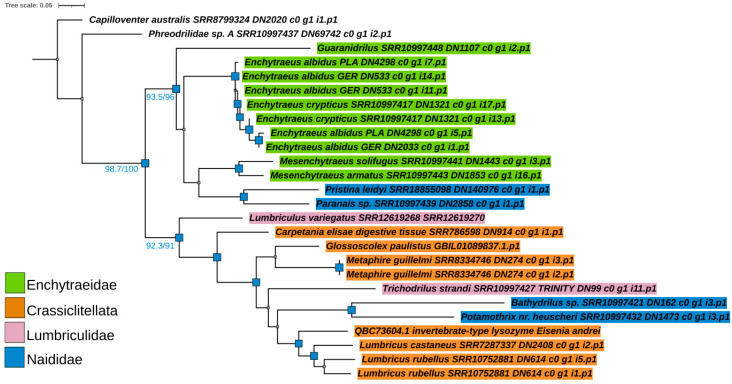
The best-scoring maximum likelihood tree (lnL = −3611.212) resulting from the analysis of mature amino acid sequences of the putative digestive i-type lysozyme in Clitellata. Only closely homologous sequences, distinct from those of other i-type lysozymes/destabilases, were used in the analysis. These sequences were retrieved from SRA raw transcriptomics data assembled in Trinity. A reciprocal BLASTp search for queries (Supplementary Table S2) revealed a match with the digestive i-type lysozyme from *Eisenia andrei* (Acc. QBC73604), with an E-value lower than 2 × 10^−90^. This sequence was used as the reference. Blue squares denote branches with both SH-aLRT and UFBoot values (if ≥70) at the respective nodes. The exact values for the selected branches are given below. The tree was rooted at *Capilloventer australis*.

Phylogenetic analysis of the putative digestive i-type lysozyme proteins found across Clitellata reveals that the tree (Figure 2) bifurcates into two distinct clades when rooted at *C*. *australis*. The first clade is composed of a single sequence from a member of the family Phreodrilidae. The second clade, which is almost maximally supported, encompasses the remaining sequences from all other analyzed clitellate families. This clade is further divided into two clusters, each receiving very high support. The first cluster contains all Enchytraeidae, with two members of Naididae (*Pristina* and *Paranais*) nested within it. These Naididae members were recovered as a sister group to *Mesenchytraeus* spp., although this relationship within Enchytraeidae is supported with low confidence. Interestingly, in a highly resolved subclade containing *E*. *crypticus* and *E*. *albidus*, the sequences were not sorted in a species-specific manner.

The second cluster consists of members of Lumbriculidae, Crassiclitellata, and two members of Naididae (*Bathydrilus* and *Potamothrix*), which are grouped mostly in a non-family-specific and highly discordant manner. Interestingly, these two naidid species belong to the subfamilies Phallodrilinae and Tubificinae, and are therefore grouped separately from members of the same family, *Paranais* and *Pristina*, which belong to the subfamilies Naidinae and Pristininae (the latter was previously included in Naidinae). Incongruent positioning of the lysozyme from the lumbriculid *Trichodrilus strandi* within the Crassiclitellata proteins, and not with *Lumbriculus variegatus*, might be attributed to low sampling of the Lumbriculidae. In contrast, the separate grouping of lysozyme sequences from different members of Naididae suggests a rather complex evolutionary history of lysozyme proteins in the family.

Based on the performed phylogenetic analysis, digestive i-type lysozyme proteins in Clitellata can be divided into three groups: (1) Capilloventridae–Phreodrilidae (as sequences from these two families were grouped together before rooting the tree), (2) Enchytraeidae–Naididae I, and (3) Crassiclitellata–Lumbriculidae–Naididae II.

### 2.6. Sequence Analysis, Domain Architecture, and Three-Dimensional Models of Selected Glycosidases

To elucidate the functional implications of the phylogenetic relationships, we performed a detailed structural analysis of the glycosidases, focusing on endo-β-1,4-glucanases (EC 3.2.1.4) and the digestive i-type lysozyme (3.2.1.17).

#### 2.6.1. Digestive i-Type Lysozyme (Ealb-iLys)

**Figure 3:**
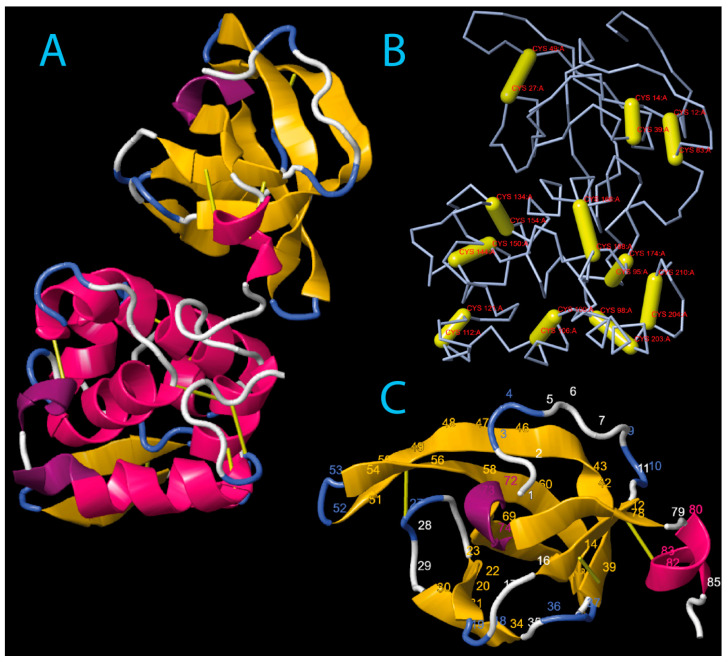
Three-dimensional model of mature digestive i-type lysozyme Ealb-iLys from the *E*. *albidus* PL-A strain (pLDDT = 91.386): (**A**) The tertiary structure of Ealb-iLys predicted by AlphaFold2/DeepMind v0.2, with secondary structure elements visualized using the First Glance in Jmol tool (version 4.1) and the DSSP 2.0 algorithm. β-Strands are shown in yellow, α-helices in pink, 3_10_-helices in magenta, turns in blue, and regions without a defined structure in white. Disulfide bridges are indicated by thick or thin yellow rods. (**B**) The spatial location of predicted disulfide bridges within the protein backbone of Ealb-iLys. The amino acid positions that form each bond were specified. (**C**) SH3b domain isolated from the rest of the Ealb-iLys protein for clarity. The selected residue numbers were labeled for reference.

**Figure 4:**
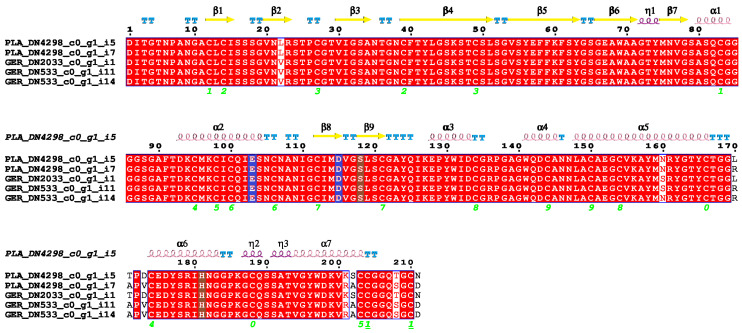
Sequence alignment and secondary structure element consensus of mature digestive i-type lysozyme Ealb-iLys allozymes from *E*. *albidus*. Secondary structure elements were predicted and marked according to Jmol using DSSP v2.0. β-Strands are marked as arrows. The α-helices and 3_10_-helices are displayed as higher and lower squiggles, respectively. The symbol η refers to the 3_10_-helix. Turns are marked as “TT” letters above the sequence. One-residue “T” segments indicate that the β-turn overlaps a structure of higher priority (e.g., a helix). The positions of potential disulfide bridges are marked as pairs of green digits below the alignment. Catalytic residues for muramidase activity are highlighted in blue, whereas those for isopeptidase activity are highlighted in brown. Strictly identical residues are shown as white characters boxed in red, while similar residues within a group are shown as red characters.

Our examination of digestive i-type lysozyme from *E*. *albidus* (referred to here as Ealb-iLys) using InterProScan and SMART uncovered the presence of an invertebrate-type lysozyme domain, commonly referred to as destabilase [57]. The classification of this protein into the subfamily GH_22i was based on the InterProScan search and the identification of the signature sequence (L/D/Y/N)SCGPYQIK, as reported by Wohlkönig and co-workers [47]. Destabilase-lysozyme proteins (i-type lysozymes) are known to have both muramidase and isopeptidase activities. The muramidase activity, typical of lysozyme, involves hydrolysis of the glycosidic bond between N-acetylmuramic acid and N-acetylglucosamine in the peptidoglycan layer of bacterial cell walls. Its function as a destabilase, an endo-ε(γ-Glu)-Lys isopeptidase, is related to the specific hydrolysis of isopeptide bonds between the γ-carboxamide group of glutamine and the ε-amino group of lysine (i.e., bonds between the side-chains of Glu and Lys) [58]. The predictive analysis identified a signal peptide of 19 amino acids, MQAAVLFVFLSV(T/A)LPAALA, with the cleavage site ALA-DIT. All pre-protein variants of Ealb-iLys were 230 amino acids long, resulting in 211 residues for the mature protein. The domain architecture of Ealb-iLys was found to encompass the destabilase-lysozyme domain and the SH3b domain (Figure 3A), the latter being easily distinguishable in the tertiary structure model as densely packed anti-parallel beta-sheets and situated upstream of the destabilase domain (Figure 3C). These two domains are linked by a short region with low compositional complexity (linker). The SH3b domain in Ealb-iLys consists of seven tightly packed beta-strands arranged as a β-barrel-like fold. The last strand is interrupted by a turn of the 3_10_ helix (η1) located between the β6 and β7 strands. The SH3b domain is zipped by the α1-helix positioned toward the C-terminal end and contains a cysteine residue that forms a potential disulfide bridge with another cysteine residue of the β1-strand (Figure 3 and Figure 4). The destabilase-lysozyme domain of Ealb-iLys consists of two parts, which can be roughly distinguished. The first part, called a subdomain, is formed by an α-helix (α2), two anti-parallel β-strands (β8 and β9) forming a β-sheet, and two relatively short α-helices (α3 and α4). This part is interconnected with another part by a long α-helix, leading to a second α-helix-based subdomain formed by two α-helices (α5 and α6) interrupted by two 3_10_-helices. Both parts of the destabilase-lysozyme domain form an active site cleft. In the destabilase from the leech *Hirudo medicinalis* (UniProt ID: Q25091), which lacks the SH3b domain in the enzyme structure, an additional 3_10_-helix is located after the first α-helix, while the β-sheet is formed by three anti-parallel β-strands, rather than two.

**Figure 5:**
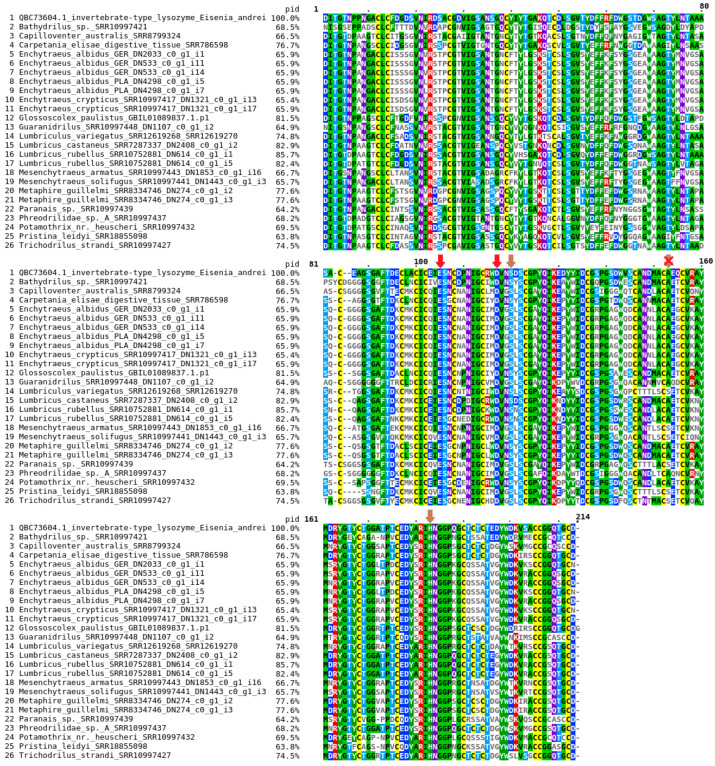
Multiple sequence alignment of i-type lysozymes containing the SH3b domain, found in Clitellata. Mature sequences of Ealb-iLys homologs were aligned. Catalytic residues for muramidase activity are marked with red arrows, whereas those for isopeptidase activity are marked with brown arrows. The crossed-out arrow indicates a semi-conserved serine previously thought to be involved in isopeptidase activity but disproven by a recent study by Marin and co-workers [57].

The conserved amino acids in Ealb-iLys for muramidase activity, glutamic acid, and aspartic acid [57] are located in the first subdomain; more precisely, Glu103 is located in the α2-helix and Asp115 in the β8-strand. In a study dedicated to a closely homologous i-type lysozyme from the earthworm *E. andrei* by Yu et al. [46], the authors mistakenly proposed a nearby serine (Ser118 in Ealb-iLys) as an additional third residue contributing to this activity. In fact, this serine is considered to be a primary candidate for the nucleophile in isopeptidase activity but not muramidase activity. Furthermore, in i-type lysozymes from mollusks, alanine often replaces a serine residue corresponding to residue 151 in Ealb-iLys [59]. This substitution is also observed in several clitellate species, including *E*. *albidus*, as we have demonstrated (Figure 5). The serine residue at this site was initially considered to be a candidate for the isopeptidase active site. However, research by Marin and co-workers [57] revealed that this residue is deeply buried within the protein core and lacks access to any protein cavities, contradicting its proposed role in isopeptidase activity.

The structural model of Ealb-iLys indicates the presence of twenty-two cysteine residues that potentially form eleven disulfide bridges (Figure 3B). Within the SH3b domain, three disulfide bridges are expected to be formed. On the other hand, the destabilase domain is predicted to contain eight bridges, which is one more (an extra one at the C-terminal end) than in the *H*. *medicinalis* destabilase. Comparative analyses with homologs of Ealb-iLys from other clitellate species (see Figure 5) spotlight two additional conserved cysteines (positions 192 and 194) in a majority of these species. This includes the Ea-iLys sequence from *Eisenia andrei*. Homology-based modeling of Ea-iLys with the AlphaFold-predicted Ealb-iLys model as a template revealed that these two cysteines can form an additional, twelfth disulfide bridge. However, the formation of this bond was the only one not favored by Disulfide by Design 2.0 analysis [60]. Nevertheless, as with many other lysozymes [59,61], the results suggest that all twenty-two cysteine residues in Ealb-iLys are involved in the formation of disulfide bonds.

#### 2.6.2. Endo-β-1,4-Glucanase I/II

Both identified endoglucanases (EC 3.2.1.4), referred to here as Ealb-Eg I and Ealb-Eg II, have been classified as members of GH family 9. We initially distinguished between these two putative genes based on their signal peptide sequences and distinct cleavage sites. However, this distinction might be somewhat oversimplified, as we identified groups of transcripts with three different open reading frame (ORF) lengths (1371, 1368, and 1353 bp), and there are no available supportive genomics data for *E*. *albidus*. Notably, the Ealb-Eg I variants from the N-strain exhibited a unique deletion of a single amino acid in the sequence, in addition to substitutions. Despite these differences, all Ealb-Eg proteins share a relatively high level of amino acid identity and possess conserved amino acid stretches that are common across variants of both genes. The pairwise sequence divergence between Ealb-Eg I and Ealb-Eg II was estimated to range from 4.1% to 30.1% (Table 7). It is also worth mentioning that the original TransDecoder-predicted longest open reading frame (ORF) for Ef-Eg I contains two additional start codons within the same frame as the coding sequence, i.e., upstream start codons within an upstream open reading frame. The proper codon site within the longest ORF was identified based on the Kozak sequence (AAC**ATG**A) variant for Annelida, as reported by Satake and coworkers [62]. This identification was further confirmed through signal peptide sequence analysis in SignalP 6.0. Notably, this Kozak sequence variant is also found in previously characterized *E*. *albidus* α-amylases [39]. Conversely, a slightly distinct ATG flanking motif (AAT**ATG**A) was identified in Ef-Eg I from the German strain. 

Because our phylogenetic analysis found that Ealb-Eg I and Ealb-Eg II proteins form a highly resolved single clade rather than separate gene-specific clusters (Figure 1), we calculated the omega (dN/dS) ratio collectively for all mature sequences of Ealb-Eg as if for a single gene. We estimated the ratio to be 0.21620, indicating that endo-β-1,4-glucanases in *E*. *albidus* are under purifying selection. Therefore, changes in their coding sequences could be detrimental. 

**Figure 6:**
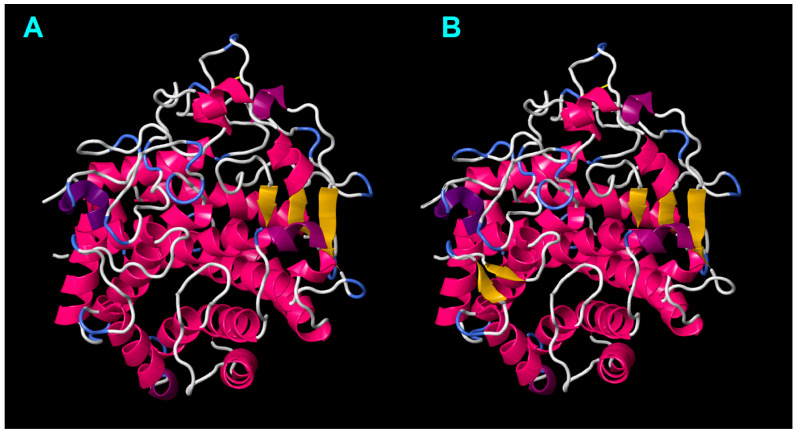
Three-dimensional models of mature endo-β-1,4-glucanases from the *E*. *albidus* PL-A strain generated by SWISS-MODEL: (**A**) Tertiary structure of Ealb-Eg I. (**B**) Tertiary structure of Ealb-Eg II. β-Strands are shown in yellow, α-helices in pink, 3_10_-helices in magenta, π-helices in purple, turns in blue, and regions without a defined structure in white. Disulfide bridges are indicated by thin yellow rods. The quality of the generated models for Ealb-Eg I/II was high, with a Global Model Quality Estimate (GMQE) of 0.92/0.93 and a QMEANDisCo global score of 0.89.

**Figure 7:**
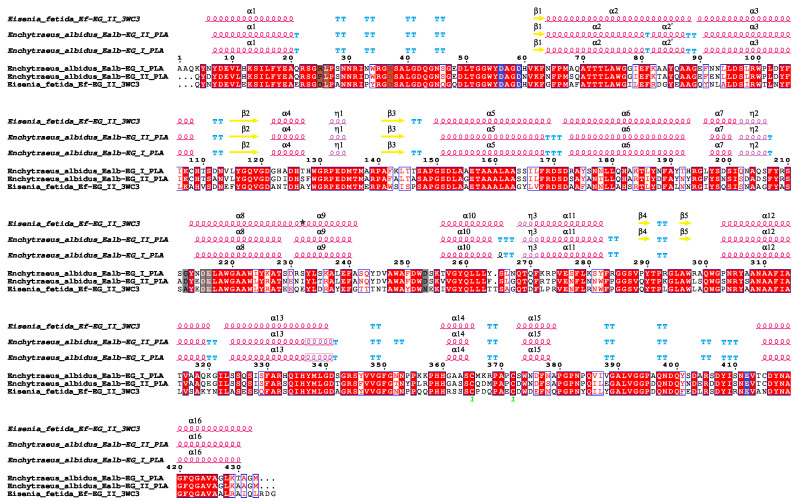
Secondary structure alignment of mature endo-β-1,4-glucanases: Ealb-Eg I/II from *Enchytraeus albidus* PL-A and Ef-EG2 from the earthworm *Eisenia fetida*. The secondary structure elements of Ealb-Eg I/II were predicted and marked according to Jmol with the implementation of the DSSP v2.0 algorithm. The secondary structure elements of Ef-EG2 were annotated according to the updated model (version 1.2) of the crystal structure of endo-1,4-beta-glucanase (PDB ID: 3WC3) from *E*. *fetida*. β-Strands are marked as arrows. The α-helices and 3_10_-helices are displayed as higher and lower squiggles, respectively. The η symbol refers to a 3_10_-helix. The boxed squiggle refers to the π-helix. Turns are marked with the letters “TT” above the sequence. One-residue “T” segments indicate that the β-turn overlaps a structure of higher priority (e.g., a helix). The position of a potential disulfide bridge is marked as a pair of green digits below the alignment. A selected residue with an alternate conformation is marked above with a black star on top of the secondary structure element annotation. Catalytic residues for cellulase activity are highlighted in blue. Residues involved in binding calcium are highlighted in gray, and those potentially involved in binding sodium are highlighted in brown. Strictly identical residues are shown as white characters boxed in red, while similar residues within a group are shown as red characters.

The domain arrangements of Ealb-Eg I and Ealb-Eg II were typical of other known GH9 endo-β-1,4-glucanases. The catalytic domain structure of Ealb-Eg proteins consists of 12 α-helices that form the (α/α)_6_-barrel fold, with six internal and six external α-helices. Additionally, the overall structure includes four extra α-helices and three conserved 3_10_-helices. Furthermore, Ealb-Eg II, similar to Ef-EG2 from the earthworm *Eisenia fetida* [63], contains five β-strands arranged as a conserved β-sheet and β-hairpin. In contrast, Ealb-Eg I lacks a β-hairpin in its structure (Figure 6 and Figure 7). The significance of this modification of the structure is not known. In both modeled Ealb-Eg proteins, a single π-helix was predicted to be located at the end of the longer α13-helix. Nonetheless, Ealb-Eg I/II proteins were found to be very similar in structure to Ef-EG2, which allowed for homology-based modeling and the generation of high-quality models.

The catalytic domains of Ealb-Eg proteins, consistent with other GH9 endo-β-1,4-glucanases, have two catalytic Asp residues within the conserved motif Asp-Ala-Gly-Asp (DAGD; here corrigendum for [64]) and one Glu residue within the semi-conserved motif Asn-Glu-Val [64], adjacent to the highly conserved Asp-Tyr-Asn-Ala (DYNA) motif of the α16-helix (see Figure 7). The study of the crystal structure of Ef-EG2 from *E*. *fetida* [63] underpins that there are binding sites for calcium and sodium ions. These sites exhibit limited conservation in *E*. *albidus* Ealb-Eg I/II and hint at a nuanced evolutionary adaptation of Ealb-Eg enzymes in ion binding, potentially reflecting distinct environmental contexts. This was observed in other GH9 endo-β-1,4-glucanases, including the enzyme from the higher termite *Nasutitermes takasagoensis* [65] (see also Supplementary Figure S1).

## 3. Discussion

### 3.1. General Considerations Regarding Digestive Enzyme Gene Candidates in E. albidus

In animals, most digestive enzymes belong to hydrolases [33,66,67,68]. Their primary function is to break down larger molecules from food into a form that can be absorbed by the organism [69]. These enzymes can be secreted into the lumen of the alimentary tract or bound to the microvilli [70]. Secreted proteins generally require a signal peptide sequence for proper targeting and secretion, whereas enzymes in microvilli have transmembrane domains that bind them to the plasma membrane or are clustered on the cell surface, requiring specific signals for proper localization and GPI anchoring [70,71,72,73]. These facts seem to have been overlooked by other authors when predicting digestive capacity based on transcriptomics data and functional annotation (see [29,30]). To distinguish intracellular metabolic and lysosomal enzymes from extracellular-acting digestive enzymes [74] in our datasets, we thoroughly analyzed the sequence features mentioned above, along with other features, in the recovered candidates for digestive enzyme genes. Among the hydrolytic enzymes, glycosidases play a crucial role in the digestion of saccharides. They are responsible for breaking down common biopolymers such as cellulose, chitin, and starch, which are abundant in nature. Glycosidases are significant in assessing trophic positions, as they define the digestive capabilities of animals by participating in the degradation of plant, fungal, or bacterial materials, including cell wall components, within the decomposer system. Moreover, glycosidases appear to be the best-characterized digestive enzymes in Annelida [41,45,46,48,63,75,76]. Although we identified candidates for proteolytic and lipolytic enzyme genes in *E*. *albidus*, the scope of the present study is somewhat limited, as we restricted our analysis to only the best annotated and orthologously supported candidates. Nevertheless, our findings provide initial insights into the genetics of enchytraeid digestive enzymes, which can be further expanded upon. While fibrinolytic proteases such as lumbrokinases are currently gathering some scientific attention, mainly for potential medical applications [77], digestive lipases remain very challenging to study not only in Enchytraeidae but also in the wider Annelida, as they are still a largely genetically unexplored group of enzymes. Recently, the hormone-sensitive lipase gene, which is an intracellular metabolic neutral lipase, was cloned and its expression was analyzed in the leech *Whitmania pigra* [78]. However, to the best of our knowledge, no dedicated molecular studies have focused on the typical digestive lipases in members of Annelida. Studies on potential digestive lipases in this taxon are often limited to biochemical enzyme assays. Indeed, the general activity patterns of hydrolytic enzymes, including lipases, in the digestive systems of representatives of Acanthobdellida, Branchiobdellida, and Hirudinida were studied using API ZYM tests by one of the co-authors of the present study [66].

### 3.2. Endogenous Expression of GH9 Cellulase Genes in E. albidus and Other Clitellates

In a review [18], the first author and colleagues proposed a classification of the trophic types of enchytraeids from the most commonly studied genera based on food preferences and feeding behavior reported in the available literature. According to the definition, primary decomposers in the soil food web consume plant litter prior to substantial microbial degradation [17,79]. Thus, it is presumed that primary decomposers need to produce enzymes involved in breaking down major plant cell wall components. In contrast, secondary decomposers rely on plant residues initially degraded by microflora or on microorganisms as food sources. *Enchytraeus* spp. were assigned to the secondary decomposer group, as no definitive evidence of endogenous cellulolytic capability has been provided before. Although some cellulase activity has been detected in a few studies on *Enchytraeus* spp., there has been no attempt to determine whether these cellulases originate from the potworms themselves or from microorganisms. Moreover, the results obtained by different authors using biochemical techniques were not always consistent [37,80,81,82]. For example, Nielsen [37], using enzymatic assays and chromatographic analyses, found no cellulolytic activity in *E*. *albidus*, nor in three other enchytraeid species. In contrast, Urbášek and Chalupský [81] detected very low to low cellulolytic activities in four species, including *E*. *albidus*. However, these authors clearly stated that there was no attempt to differentiate the origin of the detected enzymes. Similarly, Dash et al. [80] reported low-to-moderate cellulolytic activity in homogenates of entire specimens of *E*. *berhampurosus* and in two other tropical enchytraeid species. In addition to enzymatic assays, some ecohistological studies have been performed on *Enchytraeus* species. Reichert et al. [83] investigated the feeding behavior of *E*. *coronatus* on agar plates with air-dried *Sambucus nigra* leaves and observed signs of leaf tissue damage and consumption. They suggested that *E*. *coronatus* exhibited significant cellulolytic activity to pre-digest the leaves externally before ingestion. However, Gajda et al. [18] strongly disagreed with this conclusion. They performed similar experiments but included proper controls (plates with leaves but no animals), which were lacking in the study of Reichert et al. [83], and demonstrated clearly that the contribution of microbial activity to the maceration of the plant material on the experimental plate could not be disregarded as a possible explanation.

Considering all of the information recapitulated above, we proposed the following research hypothesis (1): *E*. *albidus* does not exhibit endogenous expression of enzyme genes from the cellulase group. However, our transcriptomics data analysis in this study identified 30 digestive gene candidates encoding glycosidases, among which we annotated cellulolytic enzymes—endo-β-1,4-glucanases (EC 3.2.1.4). Therefore, this hypothesis was rejected. Phylogenetic and in silico structural analyses revealed that *E*. *albidus* endo-β-1,4-glucanases are homologous to a few previously described endo-β-1,4-glucanases (cellulases) from earthworm species such as *Metaphire hilgendorfi*, *Eisenia fetida*, and *E*. *andrei* [40,41,42]. Moreover, transcriptomics data derived from other clitellate species and integrated into phylogenetic analysis demonstrated that, in addition to the aforementioned earthworm species, which provided initial evidence for endogenous cellulase production in clitellates, GH9 endo-β-1,4-glucanases are present in other members of Clitellata, including Capilloventridae, Phreodrilidae, Naididae, Lumbriculidae, and Randiellidae. Endo-β-1,4-glucanases were found to be especially widespread in members of Enchytraeidae and Crassiclitellata (i.e., earthworms). However, as a side note, it should be mentioned here that the recovered sequence for Randiellidae should be treated with caution, as the only available raw RNA-Seq reads for *Randiella* seem to be contaminated, at least to some degree, by other annelid sequences, as noted in our paper related to amylases (for details, see [39]), and this might be further indicated by the unusual result that we noticed in another study using the same transcriptomics data (please note the extraordinarily high number of linker genes in *Randiella* across all analyzed species for hexagonal bilayer hemoglobin in [84]). Apart from clitellates, we also recovered a closely homologous endo-β-1,4-glucanase from the terrestrial polychaete *Hrabeiella periglandulata*. Orthologous sequences for other polychaetes are available for the nereids *Perinereis brevicirris* and *Perinereis aibuhitensis*. Generally, all of these GH9 endo-β-1,4-glucanases from both Clitellata and Polychaeta share high similarity (≥68%) and a similar length of mature protein sequences (>420 amino acids; see also [64]). In light of this, we question the short sequence for *E. andrei* “cellulase 2” reported by Kim et al. [85], as the provided sequence lacks a signal peptide, an α1-helix in its structure and, importantly, the two catalytic Asp residues in the DAGD motif, which are essential for cellulase activity. The provided sequence for “cellulase 2” represents a 5′ partial ORF recovered from RNA-Seq data. This also underscores the importance of basic structural modeling in similar studies.

Endo-β-1,4-glucanases belonging to glycosyl hydrolase family 9 are present among diverse invertebrate lineages, demonstrating varied feeding strategies [64]. Unlike their counterparts in microbes and plants, where these cellulases often possess catalytic domains linked to carbohydrate-binding modules (CBMs) enabling crystalline cellulose breakdown [86], many GH9 animal cellulases lack such CBMs (but cf. [87,88,89]). As a result, these enzymes exhibit limited or no activity against crystalline cellulose but break down the amorphous fraction of the polysaccharide. Consequently, Linton [64] posited that the capacity to hydrolyze crystalline cellulose efficiently should serve as a proper indicator for assessing cellulases, suggesting that cellulolytic enzymes solely capable of breaking down carboxymethylcellulose (CMC) should not be considered genuine cellulases but, rather, enzymes digesting β-1,4-glucans. Additionally, it was raised that endo-β-1,4-glucanases in some animals can cleave lichenan or mixed-linkage β-D-glucans at comparable or even greater rates compared to CMC. While Linton has a point in their postulation, it is rather not universally accepted by other authors. However, based on research on other polysaccharides, it could also be argued that, for example, different amylose forms (e.g., amylose A and B) can be digested by α-amylases with extremely different efficiencies [75]. Furthermore, concerning Linton’s discussion on deriving the amounts of metabolizable sugars from cellulosic material in non-primarily herbivorous invertebrates, research on *E. fetida* demonstrated that a single amino acid substitution in the sequence can dramatically change the catalytic activity and the stability of Ef-EG2 endoglucanase mutants, impacting the amount of hydrolysis products released from CMC [76]. Moreover, screening of Clitellata transcriptomes in our study revealed that the endo-β-1,4-glucanases in the enchytraeid *E*. *albidus* and the earthworms *Lumbricus* spp., *Eisenia andrei*, and *Metaphire guillelmi* are highly polymorphic. Notably, Ef-EG1 and Ef-EG2 endo-β-1,4-glucanases in *E*. *fetida* [41,90] were originally identified as distinct genes based on cloned ORFs. However, it is likely that they actually represent allelic variants, as the differences are only related to a single nucleotide resulting in a single amino acid substitution. Support from genomics data analysis could be a solution to address this issue. Despite these minimal sequence variations, purified Ef-EG1 and Ef-EG2 proteins from the *Eisenia fetida* Waki strain [90] demonstrate significant biochemical differences between each other in terms of activity and substrate specificity, which is in agreement with the above-mentioned study of Ef-EG2 mutants [76]. In *Enchytraeus*, copy variants of endo-β-1,4-glucanases are more divergent than those in *Eisenia* spp. (see Figure 1). The adaptive significance of endo-β-1,4-glucanase polymorphisms in Clitellata could be related to broader substrate specificity; however, further molecular and biochemical studies are needed to confirm this in *E*. *albidus*.

### 3.3. Endogenous Expression of Digestive i-Type Lysozyme Gene in E. albidus and Other Clitellates

Apart from hypothesis (1), related to the absence of cellulases, we postulated hypothesis (2): that *E*. *albidus* demonstrates endogenous expression of enzyme genes engaged in the digestion of microorganisms. Thus, it was presumed that secondary decomposers, which at least partially utilize the microbial material, need to produce enzymes involved in breaking down major bacterial and fungal cell wall components, such as peptidoglycan hydrolases or chitinases. To the best of our knowledge, no studies have clearly demonstrated microphytophagous (i.e., microbivorous) behavior in *E*. *albidus* related to bacteria and fungi. However, some reports are available for other *Enchytraeus* species. The first report related to the genus was probably by Dougherty and Solberg [91], who partially succeeded in maintaining *Enchytraeus fragmentosus* under monoxenic conditions with *Escherichia coli* growing on a nutrient agar medium, but the growth of the animal was suboptimal. Subsequently, Brockmeyer et al. [92] demonstrated the use of microbial protein from radiolabeled ^35^S-enriched *Bacillus cereus* and the yeast *Saccharomyces cerevisiae* for *Enchytraeus* cf. *globuliferus* and *E. christenseni* (syn. *E*. *minutus*). In relation to this, Reichert et al. [83] reported that *E*. *coronatus* fed with *B*. *cereus* was in good condition, but its reproduction rate was lower than when fed with rolled oats. The most explicit microphytophagous behavior related to bacteria and fungi has been reported for *Enchytraeus crypticus* [13,93,94,95,96,97,98]. In general, this species can use certain species of *Streptomyces* bacteria and microscopic fungi as its sole nutrient source [13,94,96]. Moreover, it can preferably consume and utilize particular species of cyanobacteria and eukaryotic microalgae [95,97].

Based on the transcriptomics data analysis of *E*. *albidus*, we identified an endogenous novel digestive i-type lysozyme, named Ealb-iLys (GH22i family; EC 3.2.1.17), and two chitinases (GH18 family; EC 3.2.1.14), referred to here as Ealb-Chit I and Ealb-Chit II. The latter enzymes will be addressed in detail elsewhere, in a separate paper. Consequently, hypothesis (2), regarding the production of enzymes involved in breaking down major bacterial and fungal cell wall components in this enchytraeid species, was supported. In a previous review, several hypotheses were proposed concerning the capacity of Enchytraeidae to utilize various bacterial strains as a nutrient source [18]. Notably, the presence of β-N-acetylglucosaminidase in the intestinal epithelium of the enchytraeid *Lumbricillus lineatus*, as reported by Gelder [99], raised speculation about the potential role of this enzyme and other murein hydrolases in breaking down bacterial cell walls in the alimentary tract of enchytraeids. Indeed, some studies on invertebrates suggest that β-N-acetylglucosaminidase may be involved in digestion [67,100,101]. However, to the best of our knowledge, no contribution of typical digestive β-N-acetylglucosaminidase to microbial cell lysis has been described to date in invertebrates, at least in Annelida. Conversely, complete coding sequences for endo-β-N-acetylglucosaminidases (EC 3.2.1.96) recovered from transcriptomics data of *E*. *albidus* lack signal peptides, and putative proteins were predicted to be localized in the cytoplasm. Therefore, these are not secretory digestive enzymes released into the gut lumen that can contribute to trophic digestion in enchytraeids, despite our initial assumption based on Gelder’s results [18,99]. Another obvious, yet at the time of review [18] rather theoretical, candidate for the enzyme involved in microbes’ digestion in enchytraeids was lysozyme. A pivotal study that significantly contributed to considering this enzyme was the identification and histolocalization of a novel digestive lysozyme, Ea-iLys, from *E. andrei* by Yu and co-workers [46]. The annotation of a homologous sequence in *E*. *albidus* posed challenges owing to the absence of a functional ortholog for this lysozyme in the KEGG database. Therefore, we initially recovered the homologous sequences based on the presence of a signature sequence for the i-type lysozymes. Additionally, the assignment of the Ealb-iLys sequence as a lysozyme had a low positive predictive value (PPV) from the PANNZER2 annotation, highlighting the significance of annotating data using diverse methods and tools. Animal lysozymes containing the SH3b domain, such as Ealb-iLys, have rarely been identified. The i-type lysozyme, which contains a destabilase with the SH3b domain, was reported as HcLyso4 in the triangle-shell pearl mussel (*Hyriopsis cumingii*), while the SH3b domain was also noted after alignment in the sequence of MGL-2 lysozyme (Acc. AB298451) from the Mediterranean mussel (*Mytilus galloprovincialis*) [61]. Additionally, we identified this domain in the above-mentioned Ea-iLys from *E*. *andrei* [46], as it was not initially annotated in the original study. Moreover, we recovered closely homologous (orthologous) sequences to *Enchytraeus*–*Eisenia*-type lysozyme containing the SH3b domain from transcriptomics data related to several clitellates, including other enchytraeid species (*Enchytraeus crypticus*, *Mesenchytraeus solifugus*, *M*. *armatus*, and *Guranidrilus* sp.). Notably, the RNA-Seq reads (SRR786598) associated with the earthworm *Carpetania elisae* (now *C. matritensis*) [102], where we also found this novel i-type lysozyme, originated from a sample consisting of isolated digestive tissues. This finding aligns with the observation that Ea-iLys is highly expressed in the gut epithelium [46]. The possible role of the SH3b domain in this type of lysozyme may be related to peptidoglycan recognition and bacterial cell wall binding [103]; however, further studies are required to confirm this hypothesis. Based on the findings presented, we propose orthologs of *Enchytraeus*–*Eisenia*-type SH3b-domain-containing i-type lysozymes (i.e., Ealb-iLys and Ea-iLys) as potential molecular markers of bacterivory in clitellates.

### 3.4. Trophic Position of E. albidus as an Intermediate Decomposer and the Status of Other Clitellates

Considering the tested research hypotheses related to the trophic position of *Enchytraeus albidus*, we found that this enchytraeid species expresses genes for both cellulases and enzymes involved in the digestion of microbial cell walls, including a specialized digestive type of lysozyme. Therefore, *E*. *albidus* combines traits of both primary and secondary decomposers and can be defined as an intermediate type of decomposer. The term “intermediate decomposers” was originally coined by Eisenhauer and Schädler [104] to roughly define the position of enchytraeids and highlight the uncertain trophic position of this taxon, which could represent a functional gradient ranging from primary to secondary decomposers. Our transcriptomics approach, novel to trophic ecology studies, in which we determined *E*. *albidus* as an intermediate decomposer, corresponds well with the newest findings related to *E*. *albidus* sensu lato by Korobushkin et al. [105] using stable isotope analysis. In that most recent study (note: published when our manuscript was under review), where trophic niches of 16 common terrestrial enchytraeid species were determined, the analysis found them to act as primary and secondary decomposers within three trophic guilds (epigeic, epi-endogeic, and endogeic), depending on species. Korobushkin et al. [105] assigned epigeic enchytraeids, including *E*. *albidus* sensu lato (identified based on morphology only), among primary decomposers feeding on litter. However, the wide ranges of Δ^15^N values obtained in the study also indicated the co-ingestion of microorganisms. Thus, the revealed trophic niche of *E*. *albidus* matches with intermediate decomposers. Furthermore, Korobushkin et al. [105] expressed the view that the classification of individual enchytraeid species as primary or secondary decomposers requires further experimental intervention, incorporating multiple metrics instead of solely relying on stable isotopic signatures. We believe that the presented transcriptomics approach could provide a solution to this challenge.

The composition of digestive enzyme genes in *E*. *albidus* revealed by transcriptomics analysis is in general agreement with the results of the study by Urbášek and Chalupský [81], who analyzed enzymatic profiles from the whole-body homogenates of enchytraeids. *Enchytraeus albidus* was characterized there by moderate activity of α-amylase, β-xylanase, laminarinase, and lichenase, and low to very low activity of proteases (pH = 6.0), Cx-cellulase (endo-1,4-β-D-glucanase, EC 3.2.1.4), and the cellulase complex (a mixture of exo- and endo-1,4-β-D-glucanases). Moreover, our study revealed homologous sequences for conserved cellulases and digestive i-type lysozymes in the transcriptomics data of other clitellates, particularly for enchytraeid and earthworm species, suggesting a similar trophic position of these animals. However, recent work by Korobushkin et al. [106] using stable isotopes demonstrated that the trophic position of enchytraeids and earthworms can differ based on available food sources. In their microcosm experiment, they observed that enchytraeids (a mixture of littoral species, *E*. *albidus* sensu lato, and *Lumbricillus* spp.) were preconditioning the macroalgal material, while probably grazing on bacteria as well, making it suitable for the earthworm *Eisenia fetida*, which lacked direct feeding activity on non-conditioned macroalgae. This indicates that marine littoral enchytraeids can act as primary/intermediate decomposers, while *E*. *fetida* serves as a typical secondary decomposer in this specific scenario, depending on food availability. The results of the study by Korobushkin et al. [106] are in contrast to other research that considered earthworm species such as *Lumbricus terrestris* as primary decomposers in soil microcosm experiments while assigning enchytraeids to a higher trophic level [107]. Interestingly, it was demonstrated that *L*. *terrestris* can also function as a granivore and seedling herbivore [107]. Concerning this species, we found that *L*. *terrestris* congeners possess both cellulases and digestive lysozyme, similar to enchytraeids and other earthworms. The use of an enchytraeid species mixture by Korobushkin et al. [106] prevents drawing conclusions strictly for *E*. *albidus*; however, our study demonstrated that this enchytraeid species expresses several enzymes (e.g., EC 3.2.1.6, EC 3.2.1.51, and EC 3.2.1.78) that could be potentially engaged in the digestion of macroalgal material [108,109], which could be expected from typical marine littoral species. Dietary flexibility, which is a known challenge in trophic ecology studies, could be analyzed by a comparative study of enzymes of both enchytraeid and earthworm species, as in the above example, but this requires sufficiently deep sequenced transcriptomes for all species of interest and general molecular and biochemical knowledge of digestive enzymes. In general, much work remains to be conducted on the trophic position of Enchytraeidae, as well as other clitellates and their digestive capacities. A natural progression in research would involve studying food-dependent gene expression, molecular cloning, and the utilization of expression vectors to further investigate the biochemical properties of the identified digestive enzymes. Next, a more than 60-year-old dilemma related to the feeding mechanism and exact mode of digestion in enchytraeids (pre-oral digestion or internal digestion?) [18,24,83,93,110,111,112,113,114], for which there is no consensus among researchers to date, can be analyzed by histolocalization of transcripts of selected digestive enzyme genes. Furthermore, bacterivory in deep molecular details was recently studied in the model nematode *Caenorhabditis elegans*. This includes the fate of various bacterial strains ingested, chemical cues stimulating feeding and digestion, specific lysozyme expression, signaling pathways regulating digestion of bacteria, and recognition of palatable and unpalatable food ([115,116,117]; see also [118], preprint). These studies shed new light on somewhat forgotten yet crucial preliminary studies conducted by Krištůfek et al. [94], which relate, among other things, to chemoattraction in enchytraeid–bacteria interactions and primarily demonstrated that bacteria can serve as an important source of food for enchytraeids. Finally, more advanced enchytraeid and earthworm molecular studies require support from annotated genomics datasets. The first step in this direction was performed by Amorim and co-workers [56], who provided raw but high-quality genomics data for *Enchytraeus crypticus* isolate CE2183. We hope that more genomics and monohaplotype-derived transcriptomics data will be generated for enchytraeid and earthworm species in the near future. This will significantly enhance the advancement of molecular research on the trophic ecology of these groups of clitellates.

## 4. Materials and Methods

### 4.1. Animal Material

The initial culture of *Enchytraeus albidus* was established from a stock culture purchased on the e-commerce platform Allegro from a commercial seller, Bodzio-1234. The animals were kept at room temperature in a plastic box with defaunized garden soil and fed fish flakes twice weekly. Random specimens from the initial culture underwent DNA barcoding (Acc. MK044803–MK044805) and were analyzed using PCR-RF-SSCP (PCR–restriction fragments–single-strand conformation polymorphism) [119] of the Folmer fragment (Supplementary Figure S2). A COI-monohaplotype culture (PL-A strain; Acc. MK044803) was obtained from a single cocoon transferred and hatched on a 1% molecular grade agarose plate. Juvenile specimens were then relocated to defaunized soil and maintained as described earlier. The genetic purity of the established culture was confirmed by amplifying and sequencing the COI gene fragment. 

### 4.2. RNA-Seq Data Generation for the E. albidus PL-A Strain

In the preliminary study, the number of *E*. *albidus* specimens required for obtaining an optimal amount of RNA was experimentally determined by extracting RNA from one to five specimens per sample using the GeneMATRIX Universal RNA Purification Kit (EURx, Gdańsk, Poland), following the manufacturer’s protocol. The concentration and quality of the isolated RNA were assessed using a NanoDrop 2000 (NanoDrop Technologies, Wilmington, DE, USA). Additionally, cDNA was synthesized by reverse-transcribing half a microgram of RNA, primed with oligo(dT)_20_, according to the instructions provided with the NG dART RT kit (EURx). Control PCR was conducted for proper nucleic acid purification, targeting the coding sequence of α-amylase I from *E*. *albidus* (Acc. OQ830662; [39]). Each PCR mixture, with a total volume of 50 µL, consisted of EURx Color OptiTaq PCR Master Mix (2×) (final concentration: 1.25 U OptiTaq DNA Polymerase, 1.5 mM MgCl_2_, 0.2 mM of each dNTP), 0.2 µM forward AmyStrF (5′-ATGCTGTCACTGATTGTGTTTTGTC-3′) and reverse AmyEndR (5′-TCAGACATGTAGAGCAATCATGG-3′) primers, and 1 µL of cDNA as the template. The amplification thermal profile was set as follows: an initial denaturation at 95 °C for 260 s, followed by 35 cycles of denaturation at 95 °C for 40 s, annealing at 45 °C for 45 s, and extension at 72 °C for 60 s, with a final extension at 72 °C for 120 s. To confirm amplification, the PCR products were run on a 1.2% agarose gel in TBE buffer with the addition of SimplySafe (EURx).

Adult specimens of the *E*. *albidus* PL-A strain in live form, on agarose plates, were dispatched to A&A Biotechnology (Gdańsk, Poland) for the extraction of RNA. The extraction procedure involved the use of the Total RNA Mini Kit with DNase treatment (A&A Biotechnology) and was conducted on a pooled sample of four adult specimens. The quality/concentration of extracted RNA was analyzed by agarose gel electrophoresis and by the NanoDrop 2000. To generate RNA-Seq reads, RNA samples were sent to Macrogen Europe (Amsterdam, The Netherlands) via A&A Biotechnology. The cDNA library was prepared using the TruSeq Stranded mRNA LT Sample Prep Kit (Illumina, San Diego, CA, USA). Subsequently, paired-end sequencing was performed on the Illumina platform (NovaSeq 6000; 2 × 151 bp reads).

### 4.3. Transcriptome De Novo Assembly and Data Annotation

Sequence quality control of all raw reads was performed using FastQC (https://www.bioinformatics.babraham.ac.uk/projects/fastqc/, accessed on 21 March 2024). The removal of adapters and quality trimming were executed using the BBDuk plugin in Geneious Prime version 2023.2.1. The settings used were as follows: adapter trimming (default settings), partial adapter trimming from ends with a kmer length of 11, low-quality trimming at both ends with a minimum quality of 20, and adapter trimming based on paired read overhangs with a minimum overlap of 24. It is important to note that we experimented with two quality values for trimming low-quality ends, specifically, scores of 20 or 24 using Phred33. However, we found that a quality value of 24 was overly restrictive, consequently hindering the assembly’s effectiveness in recovering some of the digestive enzyme gene transcripts.

In addition to the generated *E*. *albidus* PL-A strain transcriptomics data, we retrieved raw reads data (Illumina HiSeq 2500 runs) related to the freeze-tolerant German (G) and Greenlandic (N) strains of the same species from the NCBI Sequence Read Archive (SRA: SRP108369). Moreover, we assembled and assessed transcriptomics data available in the Sequence Read Archive (SRA) repository for other clitellates, with special emphasis on enchytraeid species.

Each transcriptome was assembled separately using Trinity RNA-Seq [120,121] integrated in the OmicsBox suite version 3.0.30 using the default k-mer length settings. Assembled transcriptomes were tested for completeness using Benchmarking Universal Single-Copy Orthologs (BUSCO) [122] analysis against the metazoan database, using a Blast e-value threshold of 1 × 10^−5^. Transcriptomes were further processed using TransDecoder (http://transdecoder.github.io, accessed on 21 March 2024) with default settings to detect coding regions. TransDecoder-predicted ORFs were translated into amino acid sequences of at least 100 amino acids in length and annotated using a combination of the GhostKOALA/KofamKOALA automatic annotation and KEGG mapping service [123,124] and PANNZER2 (http://ekhidna2.biocenter.helsinki.fi, accessed on 21 March 2024) [125]. The functional annotation included KO (KEGG Orthology) assignment, KEGG pathway mapping, and prediction of gene ontology (GO) terms. Transcriptome decontamination was carried out by removing non-animal-originating KEGG-annotated sequences with the use of the QIIME filter fasta script [126] on the Galaxy platform [127]. The obtained clean data were screened for hydrolases—more specifically, glycosidases, peptidases, and lipases.

### 4.4. In Silico Analysis of Annotated Data

The annotated sequences were analyzed by several bioinformatics tools. Sequence similarity searches were conducted using BLASTp [128]. Prediction of signal peptides was performed with SignalP 6.0 [129]. The potential subcellular localization was carried out by DeepLoc 2.0 [130] and BUSCA (Bologna Unified Subcellular Component Annotator) [131]. Transmembrane domains were predicted using DeepTMHMM [132]. Glycosylphosphatidylinositol anchoring was predicted by NetGPI 1.1 [133]. Furthermore, protein domain architectures were predicted using InterProScan [134] and SMART [135]. For glycoside hydrolase (GH) family assignment, especially in complex cases, the web server for dbCAN3, an automated carbohydrate-active enzyme and substrate annotation tool (https://bcb.unl.edu/dbCAN2/index.php, accessed on 21 March 2024), was used with at least three available run tools [136]. For lipases, an additional HMMs search in the PANTHER [137] library version 18.0 was performed.

### 4.5. Additional Data and Phylogenetic Analyses

Sequences recovered from *E*. *albidus* were supplemented with sequences obtained from the GenBank database and the SRA repository. For the latter, additional transcriptomics data were assembled de novo for other annelids, encompassing all enchytraeid species referenced in Table 1. Sequencing run IDs (SRR) used for the additional data assembly are provided in Supplementary Table S3.

Homologous sequences in GenBank were identified through a BLASTp search. The protein sequences were aligned using either MAFFT 7 [138] with an automatic assignment of the alignment strategy or MUSCLE [139], depending on the dataset. The resulting alignments were visually inspected for accuracy. A web server version of IQ-TREE was employed to estimate the best-fitting model of amino acid evolution and subsequently construct a maximum likelihood tree. All trees were built using the model suggested by IQ-TREE, with 1000 replications. Ultrafast Bootstrap (UFBoot) and SH-like Approximate Likelihood Ratio Test (SH-aLRT) support values were calculated using 1000 replicates with default settings. The generated trees were rooted according to the previously proposed phylogenetic hypothesis for Clitellata [55] and visualized using iTOL [140].

### 4.6. Sequence Analysis, Protein Modeling, Structural Alignment, and Visualization

Evolutionary divergence between sequences was assessed through the pairwise distance method with the Poisson correction model in MEGA7 [141]. The ratio of non-synonymous to synonymous substitutions (dN/dS) was computed using the CodeML program in the PAML 4.9 package [142], on the Galaxy platform [127]. The 3D structure of the proteins of interest was modeled using AlphaFold2/DeepMindv0.2 [143] on the Superbio.ai platform (https://www.superbio.ai, accessed on 21 March 2024) or via homology-based modeling using SWISS-MODEL [144]. The quality of the models was evaluated using pLDDT confidence scores and SWISS-MODEL structure assessment methods (GMQE, QMEANDisCo, and QMEAN Z-scores), respectively. Secondary structure alignments were initially created using ESPript [145] and modified according to the predicted structure by implementing the DSSP 2.0 algorithm in Jmol within FirstGlance in Jmol version 4.1 (http://firstglance.jmol.org, accessed on 21 March 2024). Figures of the tertiary structure of proteins were rendered with the same tool.

## 5. Conclusions

Based on RNA-Seq data, we identified cellulolytic enzymes (endo-β-1,4-glucanases) and enzymes engaged in the digestion of microorganisms (i-type lysozymes and two chitinases) in *Enchytraeus albidus*. Thus, *E*. *albidus* combines traits of both primary and secondary decomposers and is defined as an intermediate type of decomposer. Through phylogenetic and bioinformatic analyses, it was determined that the endo-β-1,4-glucanases in *E*. *albidus* share homology with those previously described in a few species of earthworms. These GH9 cellulases were also found in transcriptomics data of other clitellates, predominantly enchytraeids and earthworms. Closely homologous sequences to *Enchytraeus*–*Eisenia*-type destabilase-lysozyme, which contains the SH3b domain, were identified in transcriptomics data from other clitellates as well. The presence of close orthologs of the *Enchytraeus*–*Eisenia*-type SH3b-domain-containing i-type lysozyme is a potential molecular marker of bacterivory in clitellates. Our study demonstrates that RNA-Seq, even with a single sample but with sufficiently deep sequencing and taxonomically well-characterized input, could be a powerful and cost-effective tool, yet it is surprisingly rarely used in trophic ecology studies.

## Figures and Tables

**Table 1:** Transcriptomes available in the SRA repository for enchytraeid species, and their completeness.

		BUSCO				
Species	SRR Run(s) for Assembly	Complete Single-Copy	Complete Duplicated	Fragmented	Missing	BUSCO Groups Complete Representation
*Guaranidrilus* sp.	SRR10997448	7.55%	18.66%	15.72%	58.07%	26.21%
*Grania simonae*	SRR10997449	12.40%	30.30%	11.0%	46.30%	42.70%
*Mesenchytraeus armatus*	SRR10997443	16.98%	43.08%	17.72%	22.22%	60.06%
*Mesenchytraeus pedatus*	SRR10997442	10.27%	64.99%	11.43%	13.31%	75.26%
*Mesenchytraeus solifugus*	SRR10997441	12.58%	64.99%	10.38%	12.05%	77.57%
*Enchytraeus crypticus*	SRR10997417	15.93%	44.97%	18.76%	20.34%	60.90%
*Enchytraeus albidus* German strain	SRR5633671, SRR5633673, SRR5633674, SRR5633678, SRR5633679, SRR5633680	7.86%	86.48%	3.98%	1.68%	94.34%
*Enchytraeus albidus* Nuuk strain	SRR5633669, SRR5633670, SRR5633672, SRR5633676, SRR5633677, SRR5633681	7.55%	87.95%	3.04%	1.47%	95.50%
*Enchytraeus albidus* PL-A strain (this study)	SRR24185061	29.45%	67.51%	1.47%	1.57%	96.96%

**Table 2:** Comparison of the KEGG-annotated transcriptomes of different *E*. *albidus* strains. The table summarizes the number of sequences (entries) and their classification into functional categories by GhostKOALA.

	PL-A	German	Nuuk
Raw dataset entries	84,423	125,364	113,553
Clean dataset entries (after decontamination)	72,044	103,077	96,427
Clean dataset annotated entries (after decontamination)	34,412 (47.8%)	50,473 (49.0%)	48,234 (50.0%)
Protein families: genetic information processing	7451	10,394	9995
Environmental information processing	4424	6026	5953
Genetic information processing	4321	7063	6701
Protein families: signaling and cellular processes	4049	5957	5348
Cellular processes	2784	4114	3872
Protein families: metabolism	2062	2887	2824
Organismal systems	1696	2399	2361
Carbohydrate metabolism	1190	2016	1984
Human diseases	1117	1460	1493
Lipid metabolism	1030	1567	1605
Unclassified: metabolism	747	1175	1094
Glycan biosynthesis and metabolism	723	1076	974
Amino acid metabolism	698	976	925
Nucleotide metabolism	488	717	651
Unclassified: signaling and cellular processes	411	650	514
Energy metabolism	401	743	749
Metabolism of cofactors and vitamins	397	620	583
Metabolism of other amino acids	239	397	376
Metabolism of terpenoids and polyketides	86	92	111
Unclassified: genetic information processing	44	62	63
Xenobiotics biodegradation and metabolism	28	39	38
Number of assigned non-redundant KOs for metabolic enzymes	1948	1959	1962
Number of assigned redundant/non-redundant KOs for glycosidases (EC 3.2.1)	39/38	41/39	45/42

**Table 3:** Digestive enzyme gene candidates identified among annotated glycosidases from transcriptomics data of *E*. *albidus*.

	Gene Name	Enzyme Commission (EC) Number	GH Family Classification	KEGG Orthology (KO) Identifier	Recovered in Strain			Total Number of Protein Variants	Predicted Localization	Notes
					PL-A	German	Nuuk			
1	α-Amylase I	3.2.1.1	GH13_24	K01176	+	+	+	6	Extracellular	Reported in [39].
2	α-Amylase II	3.2.1.1	GH13_24	K01176	+	+	+	6	Extracellular	Reported in [39]. It might exhibit additional transglycosylation activity (EC 2.4.1.25).
3	Maltase-glucoamylase, intestinal	3.2.1.3; 3.2.1.20	GH31_1	K12047	+	+	+	6	Cell membrane	Because maltase-glucoamylase and sucrase-isomaltase share a common ancestry and striking structural similarities, an alternative EC annotation with dbCAN3 indicates sucrase-isomaltase (EC 3.2.1.48; EC 3.2.1.10).
4	Maltase-glucoamylase, intestinal-like isoform	3.2.1.20	GH31_1	K01187	Partial	+	+	7	Extracellular	
5	Maltase-glucoamylase, intestinal-like isoform X2	3.2.1.20	GH31_1	K01187	+	+	+	5	Extracellular	
6	Lysosomal α-glucosidase	3.2.1.20	GH31	K12316	+	+	+	8	Extracellular	Analyses with DeepLoc 2.0 and BUSCA are in agreement regarding the extracellular localization of the protein.
7	Endo-β-1,4-glucanase I	3.2.1.4	GH9	K01179	+	+	+	7	Extracellular	Homologous endoglucanases were reported for the earthworms *Metaphire hilgendorfi* [40], *Eisenia fetida* [41], and *E*. *andrei* [42], as well as for the polychaetes *Perinereis brevicirris* [43] and *P*. *aibuhitensis* (Acc. ANR02619).
8	Endo-β-1,4-glucanase II	3.2.1.4	GH9	K01179	+	+	+	14	Extracellular	
9	Endo-1,3(4)-β-glucanase	3.2.1.6	GH81	K01180	+	+	+	6	Extracellular	Based on transcriptomics data, complete homologous sequences were recovered for *Eisenia andrei* (SRR11091733–SRR11091735), *Lumbricus castaneus* (SRR7287337), *L*. *rubellus* (SRR10752881), and *Hrabeiella periglandulata* (SRR10997424), while a partial sequence was found for *Enchytraeus crypticus* (SRR10997417).
10	β-Glucosidase/xylosidase I	3.2.1.21; 3.2.1.37	GH3	K05349	+	+	+	10	Extracellular	Analysis using dbCAN3 revealed additional EC assignments for these proteins, i.e., EC 3.2.1.55, EC 3.2.1.6, and EC 3.2.1.73.
11	β-Glucosidase/xylosidase II	3.2.1.21; 3.2.1.37	GH3	K05349	+	+	+	7	Extracellular	
12	β-Glucosidase/xylosidase III	3.2.1.21; 3.2.1.37	GH3	K05349	Partial	+	+	3	Extracellular	
13	β-Glucosidase/xylosidase IV	3.2.1.21; 3.2.1.37	GH3	K05349	+	+	+	5	Extracellular	
14	β-Glucosidase/xylosidase V	3.2.1.21; 3.2.1.37	GH3	K05349	+	+	+	7	Extracellular	
15	Chitinase I	3.2.1.14	GH18	K01183	+	+	+	6	Extracellular	This is a homolog to a novel digestive chitinase from *Eisenia andrei* [44] and *E*. *fetida* [45].
16	Chitinase II	3.2.1.14	GH18	K01183	+	+	+	3	Extracellular	This is a divergent paralog of chitinase I that possesses an additional catalytic domain.
17	Di-N-acetylchitobiase	3.2.1.-	GH18	K12310	+	+	+	6	Extracellular	-
18	Di-N-acetylchitobiase isoform X1	3.2.1.-	GH18	K12310	+	+	+	3	Extracellular	-
19	Lysozyme (i-type)	3.2.1.17	GH22i	N/A	+	+	Partial	7	Extracellular	This is a close homolog to a novel i-type digestive lysozyme from *Eisenia andrei* (Acc. QBC73604) reported in [46]. The annotation of the destabilase domain also indicates isopeptidase (EC 3.5.1.44) activity. GH classification was assessed based on the WebLogo sequence signature from [47].
20	β-Galactosidase	3.2.1.23	GH35	K12309	+	+	+	6	Extracellular	Nielsen [37] reported β-galactosidase activity in enchytraeids and earthworms.
21	β-Glucuronidase	3.2.1.31	GH2	K01195	+	+	+	7	Extracellular	-
22	α-L-fucosidase I	3.2.1.51	GH29	K01206	+	+	+	3	Extracellular	Putative homologous sequences were identified by BLASTp in other annelids (*Owenia fusiformis*, *Ridgeia piscesae*, *Capitella teleta*), as well as in some mollusk and echinoderm species.
23	α-L-fucosidase II	3.2.1.51	GH29	K01206	+	+	+	8	Extracellular	
24	α-L-fucosidase III	3.2.1.51	GH29	K01206	+	+	+	6	Extracellular	
25	α-L-fucosidase IV	3.2.1.51	GH29	K01206	+	+	+	13	Extracellular	-
26	β-Mannosidase	3.2.1.25	GH2	K01192	Partial	+	+	8	Extracellular	-
27	Mannan endo-1,4-β-mannosidase I	3.2.1.78	GH5_10	K19355	+	+	+	5	Extracellular	-
28	Mannan endo-1,4-β-mannosidase II	3.2.1.78	GH5_10	K19355	Partial	+	+	6	Extracellular	-
29	Mannan endo-1,4-β-mannosidase III	3.2.1.78	GH5_10	K19355	Partial	+	+	8	Extracellular	-
30	Mannan endo-1,4-β-mannosidase IV	3.2.1.78	GH5_10	K19355	+	+	+	7	Extracellular	This is a close homolog to endo-1,4-β-mannanase from *Eisenia fetida* (Acc. BBB35836), reported in [48].

**Table 4:** Putative digestive fibrinolytic proteases identified among trypsin-like and chymotrypsin-like sequences from transcriptomics data of *E*. *albidus* and their in silico characterization.

	GhostKOALA-KofamKOALA Annotation			All Data	SignalP 6.0	DeepTMHMM	DeepLoc 2.0	Swiss Model			BLASTp		SMART and InterPro
	Enzyme	KO	Gene	Total Number of Protein Variants	Signal Peptide	Transmembrane Region and Topology Prediction	Subcellular Localization Prediction and Probability	Possible Template, Its Origin, and Accession Number	Identity [%]	GMQE	Hit and GenBank Accession Number	Identity [%]	Predicted Domain and Family
1	Trypsin (EC 3.4.21.4)	K01312	*PRSS1/2/3*	9	Yes	Globular + signal peptide	Extracellular (0.94)	Fibrinolytic enzyme Ej-FEI-1; *Enchytraeus japonensis*; Uniprot ID H1A7B3	83–84	0.90	Fibrinolytic enzyme *Enchytraeus japonensis* Ej-FEI-2; BAL43183	84	Tryp_SPc; Peptidase S1A, chymotrypsin family
2	Chymotrypsin (EC 3.4.21.1)	K01310	*CTRB*	14	Yes	Globular + signal peptide	Extracellular (0.95)	Fibrinolytic enzyme component B; *Eisenia fetida*; PDB ID 1ym0.1.A	48–50	0.68–0.69	Fibrinolytic enzyme *Enchytraeus japonensis* Ej-FEIII-2b; BAL43192	49	
3	Trypsin (EC 3.4.21.4)	K01312	*PRSS1/2/3*	1	Yes	Globular + signal peptide	Extracellular (0.96)	Cationic trypsin; *Bos Taurus*; PDB ID 4xoj.1.A	29	0.61	Fibrinolytic protease 0 *Eisenia fetida*; ABG68022	53	
4	Pancreatic elastase 1/2 (EC 3.4.21.36) (EC 3.4.21.71)	K01326 K01346	*CELA1/* *CELA2*	15	Yes	Globular + signal peptide	Extracellular (0.95)	Fibrinolytic enzyme Ej-FEI-1; *Enchytraeus japonensis*; Uniprot ID H1A7B3	74–86	0.87–0.90	Fibrinolytic enzymes *Enchytraeus japonensis* BAL43184, BAL43186, BAL43182, BAL43188	74–85	

**Table 5:** Putative carboxypeptidase A/B-like and aminopeptidase N gene candidates annotated for *E*. *albidus* using transcriptomics data.

	Recovered in Strain			All Data	SignalP 6.0	DeepTMHMM	DeepLoc 2.0
Enzyme and (Pre-)Protein Length	PL-A	German	Nuuk	Total Number of Protein Variants	Signal Peptide	Transmembrane Region and Topology Prediction	Subcellular Localization
Carboxypeptidase A/B-like I (502)	+	+	+	5	Yes	Globular + signal peptide	Extracellular
Carboxypeptidase A/B-like II (505)	+	+	Partial	4	Yes	Globular + signal peptide	Extracellular
Carboxypeptidase A/B-like III (467)	+	+	+	5	Yes	Globular + signal peptide	Extracellular
Carboxypeptidase A/B-like IV (431)	+	+	+	1	Yes	Globular + signal peptide	Extracellular
Carboxypeptidase A/B-like V (424)	+	+	+	2	Yes	Globular + signal peptide	Extracellular
Carboxypeptidase A/B-like VI (425)	+	+	+	12	Yes	Globular + signal peptide	Extracellular
Carboxypeptidase A/B-like VII (432)	+	+	+	6	Yes	Globular + signal peptide	Extracellular
Carboxypeptidase A/B-like VIII (422)	+	+	+	4	Yes	Globular + signal peptide	Extracellular
Carboxypeptidase A/B-like IX (446)	+	+	Partial	6	Yes	Globular + signal peptide	Extracellular
Carboxypeptidase A/B-like X (429)	+	+	+	7	Yes	Globular + signal peptide	Extracellular
Aminopeptidase N I (968)	+	+	+	5	No	Alpha TM	Cell membrane
Aminopeptidase N II (968)	+	+	+	3	No	Alpha TM	Cell membrane
Aminopeptidase N III (1006)	+	+	+	6	No	Alpha TM	Cell membrane

**Table 6:** Putative lipase gene candidates annotated for *E*. *albidus* from transcriptomics data.

GhostKOALA Annotation			Recovered in Strain			Data	SignalP 6.0	DeepTMHMM	DeepLoc 2.0	Panther	InterPro
Enzyme and Pre-Protein Length	KO	Gene	PL-A	German	Nuuk	Total Number of Protein Variants	Signal Peptide	Transmembrane Region and Topology Prediction	Subcellular Localization	Panther Hit	Predicted Domain
Bile salt-stimulated lipase I [EC 3.1.1.3 3.1.1.13] (631)	K12298	*CEL*	Partial	+	+	6	Yes	Globular + signal peptide	Extracellular	Bile salt-activated lipase	Carboxylesterase type B
Bile salt-stimulated lipase II [EC 3.1.1.3 3.1.1.13] (636; 638)	K12298	*CEL*	+	Partial	Partial	5	Yes	Globular + signal peptide	Extracellular	Bile salt-activated lipase	Carboxylesterase type B
Bile salt-stimulated lipase III [EC 3.1.1.3 3.1.1.13] (636)	K12298	*CEL*	Partial	+	+	5	Yes	Globular + signal peptide	Extracellular	Carboxylesterase	Carboxylesterase type B
Bile salt-stimulated lipase IV [EC 3.1.1.3 3.1.1.13] (638)	K12298	*CEL*	-	+	+	3	Yes	Globular + signal peptide	Extracellular	Bile salt-activated lipase	Carboxylesterase type B
Secretory phospholipase A2 [EC 3.1.1.4] (237; 236)	K01047	*PLA2G*, *SPLA2*	+	+	+	8	Yes	Globular + signal peptide	Extracellular	RH14732P	Phospholipase A2

**Table 7:** Estimates of evolutionary divergence between sequences of Ealb-Eg I and Ealb-Eg II pre-proteins. The number of amino acid substitutions per site is shown. Standard error estimates are shown above the diagonal and were obtained by a bootstrap procedure (500 replicates). Analysis was conducted in MEGA7 using the Poisson correction model and involved 21 amino acid sequences. All ambiguous positions were removed for each sequence pair. There were a total of 456 positions in the final dataset.

		1	2	3	4	5	6	7	8	9	10	11	12	13	14	15	16	17	18	19	20	21
G_DN291_c0_g1_i1_EG_I	1		0.005	0.023	0.012	0.011	0.017	0.011	0.012	0.009	0.011	0.021	0.018	0.015	0.019	0.015	0.026	0.020	0.023	0.018	0.015	0.015
G_DN291_c1_g1_i6_EG_I	2	0.013		0.023	0.012	0.011	0.017	0.010	0.013	0.011	0.009	0.021	0.017	0.014	0.019	0.014	0.026	0.019	0.022	0.017	0.014	0.015
PL-A_DN3132_c2_g1_i2_EG_I	3	0.234	0.228		0.022	0.023	0.021	0.023	0.025	0.026	0.026	0.023	0.025	0.026	0.024	0.026	0.014	0.024	0.021	0.026	0.027	0.027
N_DN166_c0_g2_i11_EG_I	4	0.066	0.061	0.226		0.009	0.017	0.006	0.017	0.015	0.015	0.020	0.018	0.011	0.017	0.013	0.025	0.020	0.023	0.017	0.015	0.016
N_DN166_c0_g2_i15_EG_I	5	0.054	0.054	0.243	0.034		0.017	0.009	0.016	0.014	0.014	0.021	0.018	0.012	0.017	0.010	0.025	0.019	0.022	0.017	0.015	0.014
N_DN1982_c0_g1_i20_EG_I	6	0.131	0.121	0.196	0.116	0.129		0.015	0.018	0.020	0.019	0.014	0.016	0.019	0.014	0.020	0.024	0.010	0.016	0.014	0.018	0.018
N_DN1982_c0_g1_i34_EG_I	7	0.054	0.045	0.229	0.016	0.036	0.099		0.015	0.014	0.013	0.021	0.018	0.011	0.017	0.013	0.025	0.019	0.022	0.016	0.013	0.015
G_DN291_c0_g1_i11_EG_II	8	0.069	0.083	0.283	0.120	0.118	0.148	0.108		0.008	0.009	0.019	0.019	0.013	0.015	0.014	0.023	0.015	0.020	0.016	0.013	0.014
G_DN291_c0_g1_i12_EG_II	9	0.041	0.055	0.289	0.105	0.093	0.174	0.093	0.027		0.005	0.021	0.017	0.012	0.017	0.011	0.024	0.017	0.020	0.015	0.011	0.012
G_DN291_c1_g1_i9_EG_II	10	0.055	0.041	0.283	0.100	0.093	0.164	0.083	0.041	0.013		0.020	0.016	0.011	0.016	0.011	0.024	0.016	0.020	0.014	0.010	0.011
PL-A_DN9119_c0_g1_i1_EG_II	11	0.201	0.196	0.237	0.193	0.204	0.086	0.196	0.156	0.182	0.177		0.011	0.019	0.013	0.019	0.022	0.012	0.015	0.014	0.019	0.018
PL-A_DN9119_c0_g1_i5_EG_II	12	0.143	0.133	0.269	0.148	0.153	0.128	0.151	0.156	0.125	0.115	0.055		0.016	0.016	0.016	0.024	0.015	0.018	0.011	0.016	0.015
N_DN166_c0_g2_i14_EG_II	13	0.100	0.091	0.289	0.060	0.062	0.164	0.057	0.081	0.067	0.057	0.164	0.120		0.013	0.006	0.022	0.017	0.019	0.012	0.010	0.011
N_DN166_c0_g2_i26_EG_II	14	0.161	0.151	0.260	0.141	0.133	0.081	0.133	0.100	0.125	0.115	0.081	0.123	0.076		0.014	0.020	0.009	0.014	0.013	0.013	0.013
N_DN166_c0_g2_i6_EG_II	15	0.098	0.093	0.286	0.076	0.045	0.172	0.074	0.088	0.064	0.060	0.172	0.123	0.016	0.083		0.022	0.017	0.019	0.013	0.011	0.009
N_DN166_c0_g2_i9_EG_II	16	0.277	0.272	0.081	0.277	0.260	0.234	0.269	0.229	0.237	0.232	0.209	0.243	0.204	0.177	0.204		0.020	0.015	0.021	0.024	0.023
N_DN1982_c0_g1_i11_EG_II	17	0.172	0.161	0.254	0.172	0.164	0.052	0.159	0.100	0.125	0.115	0.067	0.108	0.120	0.041	0.128	0.188		0.012	0.011	0.014	0.015
N_DN1982_c0_g1_i22_EG_II	18	0.223	0.209	0.193	0.223	0.212	0.113	0.209	0.177	0.185	0.172	0.100	0.141	0.159	0.091	0.159	0.108	0.071		0.014	0.018	0.017
N_DN1982_c0_g1_i29_EG_II	19	0.138	0.123	0.274	0.123	0.130	0.093	0.110	0.118	0.103	0.088	0.100	0.060	0.064	0.071	0.081	0.196	0.052	0.088		0.010	0.011
N_DN1982_c0_g1_i30_EG_II	20	0.108	0.093	0.301	0.093	0.096	0.138	0.086	0.079	0.064	0.050	0.153	0.110	0.045	0.071	0.062	0.232	0.081	0.143	0.050		0.007
N_DN1982_c0_g1_i32_EG_II	21	0.105	0.096	0.298	0.110	0.079	0.146	0.103	0.096	0.071	0.062	0.153	0.105	0.052	0.069	0.036	0.220	0.098	0.133	0.057	0.025	

## Data Availability

The data underlying this article are available in Supplementary Materials. Raw RNA-Seq reads generated for a series of studies, including this study, were deposited in the Sequence Read Archive (SRA) at NCBI under accession number SRR24185061. Details are available under BioProject PRJNA956247. Endo-β-1,4-glucanase and destabilase-lysozyme coding sequences predicted for the *E*. *albidus* PL-A strain from RNA-Seq data were deposited in the GenBank database under accession numbers PP480665 (Ef-Eg I), PP484683-PP484684 (Ef-Eg II), and PP488544-PP488545 (Ealb-iLys).

## Supplementary Materials

The following supporting information can be downloaded at: https://www.mdpi.com/article/10.3390/ijms25094685/s1.
