# Supplementary material for: Trophic Position of the White Worm (Enchytraeus albidus) in the Context of Digestive Enzyme Genes Revealed by Transcriptomics Analysis
Source: Int J Mol Sci. 2024 Apr 25;25(9):4685. doi: 10.3390/ijms25094685 (PMC11083476; doi:10.3390/ijms25094685)
Supplement: Supplementary file 1 [file ijms-25-04685-s001.zip › Supplementary Figure S2. PCR-RF-SSCP analysis of the COI barcodes.pdf]

PCR-RF-SSCP analysis of the COI barcodes

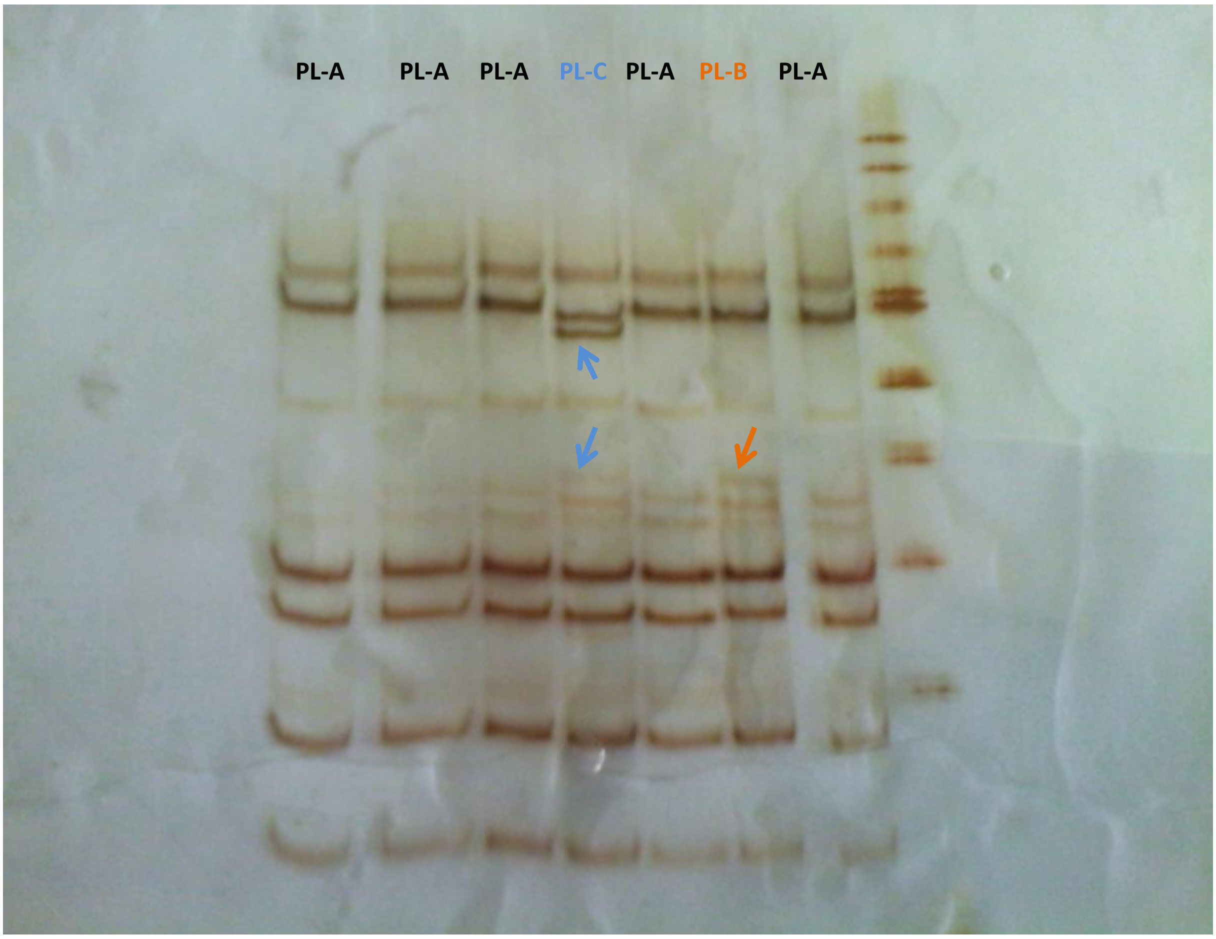

PCR-RF-SSCP gel treated with silver stain. Different haplotypes are characterized by unique SSCP pattern. Perfect 100-1000 bp DNA ladder (EURx) was used as the position marker for the bands.

PCR-RF-SSCP analysis was performed according to the protocol adapted from Rakus et al. (2008) with some modifications. The enzyme HpyCH4V, that has a restriction site (5'…TG^CA…3') was chosen to digest the COI barcodes into four fragments of 225, 204, 164, and 116 bp in size. The restriction pattern has been predicted based on consensus sequence for COI haplotypes discovered from the culture using Gene Runner 6.5.52 (http://www.generunner.net). Ten microliters of PCR amplifications were digested with 1U of HpyCH4V (New England Biolabs) in the presence of 1.5 µl of the corresponding CutSmart NEBuffer (10×), and water in total volume of 15 µl at 37 °C for 24 hours. The reaction was terminated by incubating at 65°C for 20 min. After digestion, 7.5 µl of the reaction mixture was mixed with 14 µl of SSCP loading buffer (95% formamide, 0.1% bromophenol blue, 0.1% xylene cyanole FF, 1 mM EDTA, 10 mM NaOH). Samples were denatured at 95 °C for 10 min and after that immediately cooled on ice, and loaded on 0.7 mm thick 9% polyacrylamide gel containing 5% of glycerol. Electrophoresis was performed in 1 × TBE buffer under following conditions: pre-electrophoresis (without samples) - 100V for 15 min, initial electrophoresis - 100V for 15 min, and next 600 V for 2 h 15 min, at 5 °C. In order to carry out silver stain after electrophoresis, the gel was treated with 10% ethanol for 15 min, 1% Nitric acid for 10 min, 0.01 M silver nitrate with freshly added formalin (1000 µl per liter) for 30 min, and briefly rinsed with deionized water between each step. Next, the gel was treated with small portions of freshly prepared 3% sodium carbonate with the addition of 500 µl of formalin per liter until the SSCP bands were developed and clearly visible. Finally, the gel was fixed in 10% acetic acid for 15 min and photographed.

Reference

Rakus KŁ, Wiegertjes GF, Adamek M, Bekh V, Stet RJ, Irnazarow I. Application of PCR-RF-SSCP to study major histocompatibility class II B polymorphism in common carp (*Cyprinus carpio* L.). Fish Shellfish Immunol. 2008 Jun;24(6):734-44. doi: 10.1016/j.fsi.2007.11.015
