# Supplementary figures and images for: Trophic Position of the White Worm (Enchytraeus albidus) in the Context of Digestive Enzyme Genes Revealed by Transcriptomics Analysis
Source: Int J Mol Sci. 2024 Apr 25;25(9):4685. doi: 10.3390/ijms25094685 (PMC11083476; doi:10.3390/ijms25094685)

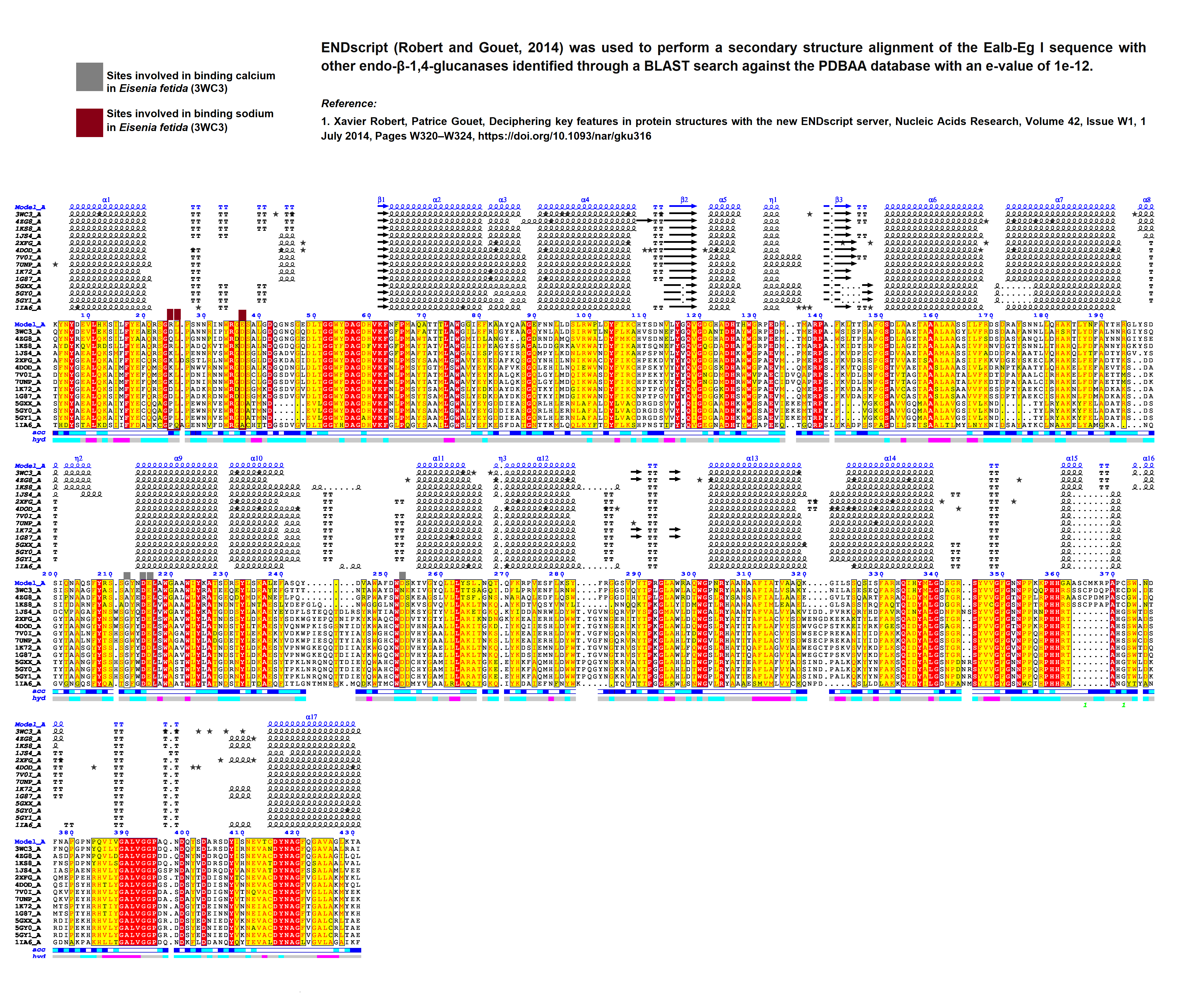

Supplement: Supplementary file 1 [file ijms-25-04685-s001.zip › Supplementary Figure S1. ENDscipt secondary structure alignment of the Ealb-Eg I with other endo-beta-1,4-glucanases.tif]
